# Supplementary material for: Growth and neurodevelopmental outcomes of preterm and low birth weight infants in rural Kenya: a cross-sectional study
Source: BMJ Open. 2023 Aug 31;13(8):e064678. doi: 10.1136/bmjopen-2022-064678 (PMC10476111; doi:10.1136/bmjopen-2022-064678)
Supplement: Supplementary data [file bmjopen-2022-064678supp001.pdf]

Supplemental Table 1. Anthropometric measurement procedures

Assessment	Methods
Weight	• Digital baby weighing scale calibrated using Standardization weight (10 kg Stone) every morning<br>• Weighing scale surface disinfected before use by the next baby<br>• Weighing scale placed on flat hard surface and made stable before placing baby on<br>• Ensured all clothing removed by caregiver (socks, diapers)<br>• Baby calmed then placed on the weighing scale (sitting or recumbent), unsupported<br>• Measurement read when the scale stopped counting, to the nearest 0.1 kg
Length	• Standard Length Mat placed on a hard flat surface with caregiver as an assistant.<br>• Length Mat surface disinfected before use by the next baby<br>• Ensured all clothing removed by caregiver (socks, diapers)<br>• Caregiver brought the child to the mat and kneeling on the left side and facing the child supported the head and neck to the correct position on mat.<br>• Assessor, kneeling on the right of child, ensured child was in perpendicular position to the base of the length mat, while supporting the knees of child, making sure the shoulders level, hands at child's side, and child's buttocks touching back of length mat<br>• Assessor moved foot piece with right hand until firmly against child's heels<br>• Measurement was read to the nearest 0.1 cm<br>• Procedure was repeated up to 2 times for confirmatory measurement
Mid upper arm circumference (MUAC)	• Used the standard measuring tape that cannot be stretched<br>• With baby on caregiver's lap, assessor exposed and positioned left arm of baby to hang loosely at the side<br>• Shoulder tip identified; tape placed at midpoint and made to run along arm<br>• With elbow flexed, tape positioned on same level, tip of elbow marked and midpoint between tip of the shoulder and tip of bent elbow identified and marked<br>• Adjusting for tension and gaps, tape placed around arm at midpoint and secured using assessor's index finger and thumb at the junction where the 0 mark of the tape meets other end of tape<br>• Measurement recorded to the nearest **0.1cm** and repeated up to 2 times for accuracy, then the average recorded
Occipital Frontal Head Circumference (OFC)	• Used a standard paper measuring tape that cannot be stretched<br>• Securely wrapped tape around widest possible circumference of the head, broadest part of forehead above eyebrow, above ears and most prominent part of back of head<br>• Measurement taken three times<br>• Largest measurement to the nearest **0.1 cm** recorded

Supplemental Table 2. Neurodevelopment Assessment Tools

**Assessment**	**Description**	**Validity**
Ten Questions Questionnaire (TQQ)[31,32]	Brief, caregiver-report screener for neurologic delay or disability. Normed for ages 2 to 9 years and adapted previously for younger children[10]<br>Delay noted if caregiver concern noted on at least one question	Acceptable sensitivity for serious disability[6,33]<br>Successfully used in African contexts[6,33]
Malawi Developmental Assessment Tool (MDAT)[10,34]	Four developmental domains: Gross motor, Fine motor & performance, Language & hearing, Social<br>Developed in Malawi as culturally relevant tool for use in Africa	Excellent reliability and good validity<br>Sensitive to differences between term and preterm infants
Hammersmith Inventory for Neurologic Examination (HINE)[35,36]	Rapid, validated, structured neurologic evaluation	High predictive validity for later cerebral palsy in children from birth to 2 years of age (90% sensitivity)<br>Successfully used in several studies in Africa, as well as clinically in Kenya

Supplemental Table 3. Demographic variables eligible infants from parent study (cRCT) versus enrolled sample for follow-up study

	Parent Study (cRCT) 28-day Survivors n (%)	Follow-up Study Sample n (%)
**Neonatal factors**		
Gender		
Male	1113 (47.0)	144 (39.8)
Female	1255 (53.0)	218 (60.2)
Gestational Age (weeks)		
≥ 37*	989 (36.1)	114 (31.5)
32 to <37	1131 (41.3)	205 (56.6)
28 to <32	183 (6.7)	34 (9.4)
22 to <28	29 (1.1)	6 (1.7)
Unknown	405 (14.8)	3 (0.8)
Birthweight (grams)		
2500 – 2999**	1005 (42.3)	129 (35.6)
1500 – 2499	1282 (54.0)	218 (60.2)
1000 – 1499	74 (3.1)	10 (2.8)
500 – 999	14 (0.6)	5 (1.4)
Apgar – 5 minute		
0 to 3	6 (0.2)	1 (0.3)
4 to 6	84 (3.1)	11 (3.0)
>= 7	2286 (83.5)	332 (91.7)
Unknown	361 (13.2)	18 (5.0)
**Maternal factors**		
Age (years)		
< 19	569 (24.0)	41 (11.3)
19 to 25	1001 (42.3)	183 (50.6)
> 25	797 (33.7)	138 (38.1)
Delivery Mode		
Vaginal	2126 (77.7)	309 (85.4)
Cesarean	230 (8.4)	48 (13.3)
Unknown	381 (13.9)	5 (13.8)

* Infants ≥ 37 weeks' gestation were included only if birthweight was < 2500 grams.
** Infants 2500 – 2999 grams were included only if gestational age was < 37 weeks.
cRCT = cluster randomized control trial

Supplemental Table 4. Child Characteristics **of enrolled sample for follow-up study** by Parent Study (cRCT) Arm

	Intervention n (%)	Control n (%)	All n (%)
Weight for Age Z-score (WAZ; Underweight; valid n=343) *			
Normal	121 (51.9)	61 (55.5)	182 (53.1)
At risk	71 (30.5)	31 (28.2)	102 (29.7)
Moderate	28 (12.0)	12 (10.9)	40 (11.7)
Severe	13 (5.6)	6 (5.5)	19 (5.5)
Length for Age Z-score (LAZ; Stunting; valid n=351) *			
Normal	100 (42.2)	53 (46.5)	153 (43.6)
At risk	81 (34.2)	21 (18.4)	102 (29.1)
Moderate	34 (14.4)	27 (23.7)	61 (17.4)
Severe	22 (9.3)	13 (11.4)	35 (10.0)
Weight for Length Z-score (WLZ; Wasting; valid n=339)*			
Normal	152 (67.3)	75 (66.4)	227 (67.0)
At risk	40 (17.7)	27 (23.9)	67 (19.8)
Moderate	15 (6.6)	5 (4.4)	20 (5.9)
Severe	11 (4.9)	3 (2.7)	14 (4.1)
Overweight	7 (3.1)	3 (2.7)	10 (3.0)
Obese	1 (0.4)	0	1 (0.3)
Composite Malnutrition (Underweight/Stunted/Wasting)**			
Normal	163 (66.5)	74 (63.3)	237 (65.5)
Malnourished	80 (32.7)	43 (36.8)	123 (34.0)
Missing	2 (0.8)	0	2 (0.6)
Past Medical Illnesses (birth until study evaluation)			
Pneumonia	18 (7.4)	10 (8.6)	28 (7.7)
Diarrheal Disease	121 (49.4)	71 (60.7)	192 (53.0)
Seizures	24 (9.8)	10 (8.6)	34 (9.4)
Malaria	141 (57.6)	64 (54.7)	205 (56.6)
Serious febrile illness/meningitis	97 (39.6)	55 (47.0)	152 (42.0)
Cough for > 2 weeks	33 (13.5)	11 (9.4)	44 (12.2)
Malnutrition	5 (2.0)	3 (2.6)	8 (2.2)
Skin infections	60 (24.5)	32 (27.4)	92 (25.4)
Current Medical Illness (in past 2 weeks)			
Acute febrile illness	3 (1.2)	1 (0.9)	4 (1.1)
Gastroenteritis/dysentery	33 (13.5)	14 (12.0)	47 (13.0)
Acute Malnutrition	4 (1.6)	0	4 (1.1)
Respiratory tract infection/pneumonia	76 (31.0)	18 (15.4)	94 (26.0)
Others ***	30 (12.2)	12 (10.3)	42 (11.6)
Referred for further care	27 (11.0)	8 (6.8)	35 (9.7)

* Normal (≥-1 for WAZ and LAZ; ≥-1 to ≤ 2 for WLZ), At risk (≥-2 to <-1), Moderate (< -2 to ≥ -3), Severe (< -3). Overweight WLZ >2 to ≤ 3, Obese WLZ > 3
** Composite malnutrition includes infants who were either underweight, stunted or wasted.
*** Other illnesses included acute conjunctivitis (1), abscess (1), thrush (4), scabies (8), dermatitis (3), skin infection (18), anemia (1), convulsions (3), otitis media (1), congenital cataract (1), worm infection (1)
cRTC = cluster randomized control trial
Note: The current study was not designed to assess the impact of the intervention on these variables. These data are presented for information only.

Supplemental Table 5. Neurodevelopmental Outcomes **of enrolled sample for follow-up study** by Parent Study (cRCT) Arm

	Intervention n (%)	Control n (%)	All n (%)
Delayed by MDAT†			
Pass/Fail criteria			
Gross Motor	11 (4.5)	4 (3.4)	15 (4.1)
Fine Motor	2 (0.8)	2 (1.7)	4 (1.1)
Language	0	3 (2.6)	3 (0.8)
Personal Social	3 (1.2)	1 (0.9)	4 (1.1)
Total MDAT*	14 (5.7)	9 (7.7)	23 (6.4)
<= -2 SD from Mean			
Gross Motor	8 (3.3)	2 (1.7)	10 (2.8)
Fine Motor	8 (3.3)	3 (2.6)	11 (3.0)
Language	6 (2.5)	4 (3.4)	10 (2.8)
Personal Social	13 (5.3)	5 (4.3)	18 (5.0)
Total MDAT*	7 (2.9)	3 (2.6)	10 (2.8)
Delayed by HINE†	6 (2.5)	1 (0.9)	7 (1.9)
Neurodevelopmental Delay††	20 (8.2)	11 (9.4)	31 (8.6)
Ten Questions Questionnaire: Total with one or more concerns	61 (24.9)	21 (18.0)	82 (22.7)

† MDAT=Malawi Developmental Assessment Tool; HINE = Hammersmith Infant Neurologic Examination
†† Neurodevelopmental Delay defined as a fail on one or more of the 3 evaluation criteria, MDAT Pass/Fail, MDAT Z-score (≤ -2 standard deviations from mean) or HINE.
* NOTE: A fail score on the total MDAT can occur with a fail in any one or more subscales, thus this number does not represent the sum of children failing on the domain scores.
cRTC = cluster randomized control trial
Note: The current study was not designed to assess the impact of the intervention on these variables. These data are presented for information only.
