# Supplementary material for: Growth and neurodevelopmental outcomes of preterm and low birth weight infants in rural Kenya: a cross-sectional study
Source: BMJ Open. 2023 Aug 31;13(8):e064678. doi: 10.1136/bmjopen-2022-064678 (PMC10476111; doi:10.1136/bmjopen-2022-064678)
Supplement: Author's manuscript [file bmjopen-2022-064678.draft_revisions.pdf]

BMJ Open is committed to open peer review. As part of this commitment we make the peer review history of every article we publish publicly available.

When an article is published we post the peer reviewers' comments and the authors' responses online. We also post the versions of the paper that were used during peer review. These are the versions that the peer review comments apply to.

The versions of the paper that follow are the versions that were submitted during the peer review process. They are not the versions of record or the final published versions. They should not be cited or distributed as the published version of this manuscript.

BMJ Open is an open access journal and the full, final, typeset and author-corrected version of record of the manuscript is available on our site with no access controls, subscription charges or pay-per-view fees (http://bmjopen.bmj.com).

If you have any questions on BMJ Open's open peer review process please email info.bmjopen@bmj.com

BMJ Open

Growth and Neurodevelopmental Outcomes of Preterm and Low Birthweight Infants in Rural Kenya: a cross-sectional study

Journal:	*BMJ Open*
Manuscript ID	bmjopen-2022-064678
Article Type:	Original research
Date Submitted by the Author:	13-May-2022
Complete List of Authors:	Martin-Herz, Susanne P. ; University of California San Francisco, Department of Pediatrics<br>Otieno, Phelgona; Kenya Medical Research Institute, Center for Clinical Research<br>Nalwa, Grace; Maseno University, Department of Paediatrics and Child Health; Jaramogi Oginga Odinga Teaching and Referral Hospital<br>Moshi, Vincent; Kenya Medical Research Institute, Center for Clinical Research<br>Olieng'o Okoth, Geofrey; Kenya Medical Research Institute, Center for Clinical Research<br>Santos, Nicole; University of California San Francisco, Institute of Global Health Sciences<br>Walker, Dilys; University of California San Fransisco, Institute for Global Health Sciences and Department of Obstetrics, Gynecology & Reproductive Sciences
Keywords:	Developmental neurology & neurodisability < PAEDIATRICS, Community child health < PAEDIATRICS, NEONATOLOGY

SCHOLARONE™
Manuscripts

I, the Submitting Author has the right to grant and does grant on behalf of all authors of the Work (as defined in the below author licence), an exclusive licence and/or a non-exclusive licence for contributions from authors who are: i) UK Crown employees; ii) where BMJ has agreed a CC-BY licence shall apply, and/or iii) in accordance with the terms applicable for US Federal Government officers or employees acting as part of their official duties; on a worldwide, perpetual, irrevocable, royalty-free basis to BMJ Publishing Group Ltd ("BMJ") its licensees and where the relevant Journal is co-owned by BMJ to the co-owners of the Journal, to publish the Work in this journal and any other BMJ products and to exploit all rights, as set out in our licence.

The Submitting Author accepts and understands that any supply made under these terms is made by BMJ to the Submitting Author unless you are acting as an employee on behalf of your employer or a postgraduate student of an affiliated institution which is paying any applicable article publishing charge ("APC") for Open Access articles. Where the Submitting Author wishes to make the Work available on an Open Access basis (and intends to pay the relevant APC), the terms of reuse of such Open Access shall be governed by a Creative Commons licence – details of these licences and which Creative Commons licence will apply to this Work are set out in our licence referred to above.

Other than as permitted in any relevant BMJ Author's Self Archiving Policies, I confirm this Work has not been accepted for publication elsewhere, is not being considered for publication elsewhere and does not duplicate material already published. I confirm all authors consent to publication of this Work and authorise the granting of this licence.

For peer review only

Growth and Neurodevelopmental Outcomes of Preterm and Low Birthweight Infants in Rural Kenya: a cross-sectional study

Susanne P. Martin-Herz, MD, PhD*
Division of Developmental Medicine, Department of Pediatrics
University of California San Francisco
Box 4054, 1825 4th Street, 6th Floor, San Francisco, CA 94143
https://orcid.org/0000-0002-2474-3904

Phelgona Otieno, MBChB, MMed Paediatrics, MPH*
Kenya Medical Research Institute, Center for Clinical Research
P.O. Box 54840 00200 Off Mbagathi Road, Nairobi, Kenya
https://orcid.org/0000-0002-2927-9848

Grace Nalwa, MBChB, MMed Paediatrics
Maseno University School of Medicine, Department of Paediatrics and Child Health
Maseno, Nyanza, Kenya

Vincent Moshi, MSc.
Kenya Medical Research Institute, Center for Clinical Research
P.O. Box 54840 00200 Off Mbagathi Road, Nairobi, Kenya

Geofrey Olieng'o Okoth, Dip. CM
Kenya Medical Research Institute, Center for Clinical Research
P.O. Box 54840 00200 Off Mbagathi Road, Nairobi, Kenya

Nicole Santos, PhD
University of California San Francisco, Institute for Global Health Sciences
550 16th St, San Francisco, CA 94158
https://orcid.org/0000-0002-7517-5177

Dilys Walker, MD
University of California San Francisco, Institute for Global Health Sciences and
Department of Obstetrics, Gynecology & Reproductive Sciences
550 16th St, San Francisco, CA 94158

* Contributed equally as co-first authors

Corresponding Author: Susanne Martin-Herz, MD, PhD, Division of Developmental Medicine at University of California San Francisco, 550 16th Street, UCSF Box 4054, San Francisco, CA 94143, 415-353-2080, Susanne.MartinHerz@ucsf.edu

Running Head: Preterm and Low Birthweight Outcome in Rural Kenya

Abbreviations: preterm (PT), low birthweight (LBW), Hammersmith Infant Neurological Examination (HINE), Malawi Developmental Assessment Tool (MDAT), TQQ (Ten Questions Questionnaire)

Keywords: Preterm, Low Birth Weight, Neurodevelopment

For peer review only

ABSTRACT

Objective: Data on long-term outcomes of preterm (PT) and low birthweight (LBW) infants in countries with high neonatal mortality rates are limited, especially from community settings. The current study sought to explore growth and neurodevelopmental outcomes of PT/LBW infants from a rural community-based setting of Kenya up to 18 months adjusted age.

Design: Cross-sectional study.

Setting: Migori County, Kenya.

Participants: Four hundred ten PT/LBW infants were recruited from a cluster randomized control trial (NCT03112018) evaluating a package of facility-based quality of care interventions around the time of birth.

Outcome measures: Caregiver interviews and infant health, growth and neurodevelopmental assessments were completed at 6, 12 or 18 months $\pm$ 2 weeks. Data included sociodemographic information, medical history, growth measurements, and neurodevelopmental assessment using the Ten Questions Questionnaire, Malawi Developmental Assessment Tool, and Hammersmith Infant Neurological Examination. Analyses were primarily descriptive, and growth data were compared to national and regional Demographic Health Survey data. No alterations were made to planned data collection.

Results: The final sample included 362 PT/LBW infants. Fewer than 2% of parents identified their child as malnourished, but direct measurement revealed higher than local proportions of stunting (27% versus 26%), wasting (11% versus 4%) and underweight (17% versus 9%). Overall, 22.7% of caregivers expressed concern about their child's neurodevelopmental status. Neurodevelopmental delays were identified in 8.6% of infants based on one or more standardized tools, and 2% showed neurologic findings indicative of cerebral palsy.

Conclusions: Malnutrition and neurodevelopmental delays are common among PT/LBW infants in this setting. Close monitoring and access to early intervention programs are needed in order to help these vulnerable infants thrive.

Strengths and Limitations:

- This study explores growth and neurodevelopment of preterm and low birthweight (PT/LBW) infants up to 18 months adjusted age in Migori County, Kenya and provides important data towards better understanding of health and neurodevelopmental outcomes among these vulnerable infants at the rural, community level.
- This study demonstrates that standardized neurodevelopmental assessment tools can be locally implemented to enhance evaluation at the community-level.
- The cohort comprised largely moderate to late preterm infants with predominately normal or low birthweight, as opposed to very or extremely PT/LBW infants, and may underestimate true rates of neurodevelopmental delays or disability.

For peer review only

- The study design did not allow for direct comparison to term, normal birthweight controls, and it was not possible to investigate factors contributing to poor growth or neurodevelopmental outcomes through multivariate analyses due to sample size constraints.

For peer review only

Complications associated with preterm (PT) birth and low birthweight (LBW) contribute to 25% to 50% of all neonatal deaths and 12% of under-5 mortality worldwide.[1,2] Additionally, close to one million PT survivors experience neurodevelopmental impairments each year, and PT birth is the fifth leading cause of Disability Adjusted Life Years (DALYs) in East Africa.[3–5] However, there is a paucity of data on the long-term outcomes of both PT and LBW infants in countries with high neonatal mortality rates (NMR), particularly from community settings.[6] In countries with an NMR $\geq$ 5, global estimates suggest approximately 24.6% of PT survivors are at risk of moderate or severe neurodevelopmental impairment and 32.5% of mild neurodevelopmental disability; however, these estimates are based on only 7 datasets, all in settings with neonatal intensive units (NICU).[3]

Data from community-based PT/LBW samples in areas without NICUs are extremely limited, meaning outcomes of the majority of PT/LBW infants born in low-income countries (LMIC) are not represented in current estimates.[6,7] Three community-based, rural cohort studies from Malawi, Rwanda and Uganda exist, showing PT or LBW babies to be significantly more likely than term infants to have died between 6 weeks and 24 months adjusted age, with death rates twice as high for premature infants at 1 and 2 years than for term infants.[8–10] Survivors were more commonly wasted or underweight.[8,10] Additionally, caregivers of PT infants were significantly more likely to express concern about their child's development than caregivers of term infants; up to two-thirds of PT/LBW infants in the Rwandan sample showed developmental delays on a standardized, validated caregiver-report developmental screening tool at an average age of 22.5 months.[9,10] PT survivors were also significantly more likely to have neurodevelopmental delays on directly-administered assessments than term counterparts, with

particular deficits in the language and fine motor domains. Being underweight or malnourished was significantly associated with delays for both term and PT infants.[8,10]

In Kenya, an estimated 12% and 10.5% of births are PT and LBW, respectively.[11,12] In Migori County, where the current study took place, rates of malnutrition in children under-5 include stunting in 26.4%, wasting in 4%, and underweight in 8.6%.[13] One study from a Kenyan urban, academic center followed very LBW (VLBW, <1500g) infants for 2 years post-discharge and found 11.7% (95% CI, 6.2-17.1) had cerebral palsy, 9.2% (95% Cl 4.2-16.9) had cognitive delay, and 26.7% (95% Cl, 12.2-36.9) had functional disability.[14] However, this sample is likely not representative of rural sites that lack NICU services.

Early interventions increasingly show improvements in long-term outcomes of PT and other at-risk babies, both in high-income settings and LMIC, highlighting the need for additional studies to better understand growth and neurodevelopment of PT/LBW infants across community settings.[6,15,16] The current study leveraged the Preterm Birth Initiative Kenya (PTBi-K) cohort[11] to explore growth and neurodevelopment of PT/LBW infants up to 18 months adjusted age in Migori County, Kenya and provides data towards better understanding of health and neurodevelopmental outcomes among PT/LBW infants at the rural, community level.

MATERIALS AND METHODS:

Design. This cross-sectional study was conducted between October 2018 to May 2019 among a subset of mothers and babies previously enrolled in PTBi-K, a cluster randomized control trial (cRCT) of a package of interventions to improve quality of care during labor and the immediate postnatal period (Clinical Trials Registration: NCT03112018). The protocol and primary results of this cRCT have been published elsewhere.[11,12]

Setting. The current study was conducted in Migori County, Kenya. The county is mostly rural, has poor access to health care and has higher infant and under-5 mortality than national statistics (50 vs. 39 per 1000 live births, and 82 vs. 52 per 1000 live births, respectively).[13]

Study Participants and Sampling Strategy. Participants in the parent cRCT were identified from maternity registers. Eligible participants were LBW (<2500g at birth) or PT (gestational age <37 weeks with birthweight <3000g) infants delivered at one of 17 facilities across the county. A list of potentially eligible infants, alive at 28 days and approaching 6, 12 or 18 months ± 2 weeks of age was created, with age adjusted for preterm status if the infant was born at less than 37 weeks' gestation. Recruitment was sequential toward the goal sample size.

A priori calculation of sample size using the Cochran's method was based on the caregiver-report Ten Questions Questionnaire (TQQ) in a community-based study of PT versus term infants in Malawi.[10,17] The calculated target sample size was n=183 per age group to detect a delay prevalence of 0.139 with a power of at least 80% and median effect size of 0.3.

Procedures. Caregivers of eligible infants were contacted via phone using a standard participation invitation script was used to explain the study. Appointments were scheduled at a study facility nearest the family's home. All consent forms and questionnaires were translated and back translated from English to Kiswahili and Dholuo.

Pregnancy, birth and neonatal course data were extracted from the cRCT database and confirmed with the caregiver when possible. Assessors were blind to the child's birthweight and gestational age, and questions regarding these variables were not asked at the study visit. The sequence of assessments was: (1) caregiver interview for sociodemographic information, medical history including growth, illness, and development, and the TQQ; (2) direct neurodevelopmental assessments including the Malawi Developmental Assessment Tool (MDAT) and Hammersmith

For peer review only

Infant Neurological Examination (HINE); and (3) physical examination including anthropometric measurements. Details of anthropometric measurement standardized guidelines and the 3 neurodevelopmental assessment tools are in Supplemental Tables 1 and 2.

All assessments were conducted in a conducive environment when the child was settled and in relatively stable health and complied with health and safety procedures. The research team consisted of clinical officers and nurses, all trained in study procedures and certified to conduct neurodevelopmental assessments. Two team members were present for each assessment, with one conducting the assessment and one observing and recording findings. A pediatrician trained in all study procedures provided consultation and regular supervision.

After assessment, feedback on the child's neurodevelopment and health was given to the caregiver and their concerns addressed. Caregivers were also given information on nutrition, danger signs for common childhood illnesses, and simple games to play with their child. Children identified with any significant health or developmental concern, such as hearing impairment, acute malnutrition or neurodevelopmental delay, were referred to appropriate follow-up care customized to patient need (e.g., audiology, nutrition support), with costs of up to 4 care visits covered by the study.

Data collection was paper based, with subsequent entry into a Microsoft Access database. Double entry and verification to test for logical sequence, discrepancies and outliers was completed. Data were de-identified and stored on an encrypted server within a locked study facility. Efforts to address potential bias included sequential recruitment toward sample size goal, reporting of differences between consenting individuals and the eligible sample, similar procedures at multiple sites to reduce loss to follow-up risk that might be associated with travel to a central location, blinding of assessors as to child's birthweight and gestational age.

For peer review only

Patient and public involvement. For the larger parent study in which participants were involved, national and community advisory boards provided input on intervention priorities. Health facility providers, managers, and local authorities were involved in implementation activities and influenced the focus and content of those activities on the basis of their roles and priorities.

While caregiver participants were not involved in the design or conduct of the study, other than being a participant, findings specific to their child's data were disseminated directly to caregivers at the visit. If neurodevelopmental delays were identified, clinical referrals were made as well.

Ethical considerations. This project was approved by the Scientific and Ethics Review Unit of the Kenya Medical Research Institute (KEMRI/SERU/CCR/0104/3668) and the University of California San Francisco Institutional Review Board (UCSF IRB#: 18-25555). Written authorization was obtained from the Migori County Director of Health. Formal written informed consent procedures were completed in the preferred language of each caregiver.

Statistical Analysis. Analyses were primarily descriptive and completed using STATA Version 13.0 Stata/MP. Child medical experiences were summarized as past medical illnesses (since birth) or current medical status (within 2 weeks of the assessment). MDAT and HINE total and domain scores were calculated. MDAT scores were investigated using 2 methods. First, a child was noted to have failed the MDAT overall if they were unable to complete 2 or more items in any one domain that would be expected to be passed by 90% of the normal reference population at their age.[18] Second, developmental z-scores were calculated using the most current MDAT Scoring Application (beta test version v1.1), and scores were dichotomized as either typical (> -2 standard deviations (SD) of mean) or delayed (≤ -2 SD to mean). For the HINE, a score of < 64 was used, as this has been shown to be 98% predictive of walking at 2 years with a sensitivity of

For peer review only - http://bmjopen.bmj.com/site/about/guidelines.xhtml

85% for PT children.[19] TQQ findings were described per age group, with overall caregiver concern noted if one or more items were endorsed.

For growth, World Health Organization (WHO) child growth standards were used in calculation of z-scores as provided in the STATA igrowup package.[20] Nutritional status z-scores of weight for age (WAZ), length for age (LAZ) and weight for length (WLZ) were calculated.[21] Outcomes were categorized into normal (≥-1 for WAZ and LAZ; ≥-1 to ≤ 2 for WLZ), at risk (≥-2 to <-1), moderate (< -2 to ≥ -3) or severe (< -3). Overweight and obese were defined as WLZ >2 to ≤ 3 and WLZ > 3, respectively. A composite dichotomous malnutrition variable was created with those meeting moderate or severe criteria in at least one of the three nutritional z-score variables considered malnourished.

All available data were included in the analyses. There were few missing datapoints, and any cases of missingness for pregnancy, infant and child health characteristics are noted in Tables 1 and 2. No datapoints were missing for the MDAT or the HINE. One 12-month-old did not have a complete TQQ. Records with missing data were omitted only for each respective analysis.

RESULTS

Of 761 eligible infants, 410 (54%) consented. A total of 28 infants (7.2%) died prior to study contact, six were not assessed due to acute illness at the time of appointment, and 14 were excluded due to data mismatch. The final sample consisted of a total 362 infants (88%) with viable data of which 155, 159 and 48 were 6-, 12- and 18-month-old respectively (Figure 1). The target sample size of 193 per age group was not reached due to the parent study ending earlier than expected and a national health worker strike that particularly restricted the pool of eligible 18-month-olds.

For peer review only

Characteristics at Delivery and Immediate Postnatal Period

Most babies were female (60.2%) and moderate to late PT (88.1%, >32 weeks' gestation); over one-third had normal birthweight, and more than 90% had 5-minute Apgar scores ≥7. Sixteen percent were admitted to the newborn unit, 35.6% needed special care (i.e., oxygen, phototherapy, kangaroo mother care) in the first month of life. Half of mothers were aged 19 to 25. Most were multiparous (70.4%), and 13% of deliveries were by C-section (Table 1).

Table 1: Delivery and Immediate Postnatal Period Characteristics

Age at Assessment	6 months n (%)	12 months n (%)	18 months n (%)	All n (%)
**Neonatal factors**				
Gender				
Male	57 (36.8)	64 (40.3)	23 (47.9)	144 (39.8)
Female	98 (63.2)	95 (59.8)	25 (52.1)	218 (60.2)
Gestational Age (weeks)				
≥ 37*	59 (38.1)	45 (28.3)	10 (20.8)	114 (31.5)
32 to <37	76 (49.0)	96 (60.4)	33 (68.8)	205 (56.6)
28 to <32	17 (11.0)	12 (7.6)	5 (10.4)	34 (9.4)
22 to <28	3 (1.9)	3 (1.9)	0	6 (1.7)
Unknown	0	3 (1.9)	0	3 (0.8)
Birthweight (grams)				
2500 – 2999**	50 (32.3)	58 (36.5)	21 (43.8)	129 (35.6)
1500 – 2499	94 (60.7)	97 (61.0)	27 (56.2)	218 (60.2)
1000 – 1499	7 (4.5)	3 (1.9)	0	10 (2.8)
500 – 999	4 (2.6)	1 (0.6)	0	5 (1.4)
Apgar – 5 minute				
0 to 3	0	0	1 (2.1)	1 (0.3)
4 to 6	7 (4.5)	4 (2.5)	0	11 (3.0)
>= 7	141 (91.0)	144 (90.6)	47 (97.9)	332 (91.7)
Unknown	7 (4.5)	11 (6.9)	0	18 (5.0)
Admitted to Newborn Unit (Yes)	28 (18.1)	22 (13.8)	8 (16.7)	58 (16.0)
"Special care" in first month (Yes)	59 (38.0)	59 (37.1)	11 (22.9)	129 (35.6)
Oxygen	19 (32.2)	8 (13.6)	3 (27.3)	30 (23.3)
Phototherapy	3 (5.1)	4 (6.8)	0 (0)	7 (5.4)
Kangaroo Mother Care	52 (88.1)	56 (89.8)	10 (90.9)	118 (91.5)
**Maternal factors**				
Age (years)				
< 19	24 (15.5)	12 (7.6)	5 (10.4)	41 (11.3)
19 to 25	71 (45.8)	85 (53.5)	27 (56.3)	183 (50.6)

For peer review only

> 25	60 (38.7)	62 (39.0)	16 (33.3)	138 (38.1)
Parity				
Primigravida	51 (32.9)	46 (28.9)	10 (20.8)	107 (29.6)
Multigravida	104 (67.1)	113 (71.1)	38 (79.2)	255 (70.4)
Delivery Mode				
Vaginal	125 (80.7)	144 (90.6)	40 (83.3)	309 (85.4)
Cesarean	26 (16.8)	14 (8.8)	8 (16.7)	48 (13.3)
Unknown	4 (2.6)	1 (0.1)		5 (13.8)

* Infants ≥ 37 weeks' gestation were included only if birthweight was < 2500 grams.
** Infants 2500 – 2999 grams were included only if gestational age was < 37 weeks.

For peer review only

Compared to the eligible pool of caregivers and infants from the parent study, mothers in the current study were older on average (24.7 years vs. 23.6 years, t=3.16, p<0.005), and babies were more likely female (60.2% vs. 52.8%, χ^2=7.73, p=0.02). The two groups did not differ significantly in other key demographic variables (Supplemental Table 3).

Growth and Health

Anthropometric measurement and caregiver-reported health findings are in Table 2. The prevalence of stunting, underweight, and wasting in the study population were 27.4%, 17.2% and 3.3%, respectively. The proportions of malnutrition increased with infant age. Moderate to severe malnutrition was significantly more common in males than females (OR 2.53, 95% CI 1.62-3.97), and in babies born after multiple gestation (OR 1.72, 95% CI 1.08-2.75) or with birthweight 1500 to 2499g (OR 1.73, 95% CI 1.07-2.81).

The most common illnesses reported as ever experienced by participants included malaria (56.7%), diarrheal disease (55.2%) serious febrile illness (42.3%); and in the past 2 weeks prior to assessment, respiratory tract infections (26%).

Table 2. Child Characteristics at Time of Visit

Age at Assessment	6 months	12 months	18 months	All

	n (%)	n (%)	n (%)	n (%)
Weight for Age Z-score (WAZ; Underweight; valid n=343) *				
Normal	87 (58.4)	68 (45.3)	27 (61.4)	182 (53.1)
At risk	43 (28.9)	52 (34.7)	7 (15.9)	102 (29.7)
Moderate	13 (8.7)	20 (13.3)	7 (15.9)	40 (11.7)
Severe	6 (4.0)	10 (6.7)	3 (6.8)	19 (5.5)
Length for Age Z-score (LAZ; Stunting; valid n=351) *				
Normal	71 (46.7)	62 (40.5)	20 (43.5)	153 (43.6)
At risk	47 (30.9)	47 (30.7)	8 (17.4)	102 (29.1)
Moderate	24 (15.8)	26 (17.0)	11 (23.9)	61 (17.4)
Severe	10 (6.6)	18 (11.8)	7 (15.2)	35 (10.0)
Weight for Length Z-score (WLZ; Wasting; valid n=339)*				
Normal	107 (73.3)	89 (59.7)	31 (70.5)	227 (67.0)
At risk	22 (15.1)	38 (25.5)	7 (15.9)	67 (19.8)
Moderate	5 (3.4)	11 (7.4)	4 (9.1)	20 (5.9)
Severe	5 (3.4)	7 (4.7)	2 (4.6)	14 (4.1)
Overweight	6 (4.1)	4 (2.7)	0	10 (3.0)
Obese	1 (0.7)	0	0	1 (0.3)
Composite Malnutrition (Underweight/Stunted/Wasting)**				
Normal	111 (71.6)	100 (62.9)	26 (54.2)	237 (65.5)
Malnourished	44 (28.4)	59 (37.1)	20 (41.7)	123 (34.0)
Missing	0	0	2 (4.2)	2 (0.6)
Past Medical Illnesses (birth until study evaluation)				
Pneumonia	9 (5.8)	13 (8.2)	6 (12.5)	28 (7.7)
Diarrheal Disease	63 (40.7)	107 (67.3)	30 (62.5)	200 (55.2)
Seizures	8 (5.2)	22 (13.8)	4 (8.3)	34 (9.4)
Malaria	55 (35.5)	107 (67.3)	43 (89.6)	205 (56.7)
Serious febrile illness/ meningitis	28 (58.3)	41 (26.5)	84 (52.8)	153 (42.3)
Cough for > 2 weeks	13 (8.4)	26 (16.4)	5 (10.4)	44 (12.2)
Malnutrition	2 (1.3)	3 (1.9)	3 (6.2)	8 (2.2)
Skin infections	26 (16.8)	51 (32.1)	15 (31.3)	92 (25.4)
Current Medical Illness (in past 2 weeks)				
Acute febrile illness	3 (1.9)	1 (0.6)	0	4 (1.1)
Gastroenteritis/dysentery	21 (13.5)	20 (12.6)	6 (12.5)	47 (13.0)
Acute Malnutrition	2 (1.3)	2 (1.3)	0	4 (1.1)
Respiratory tract infection/ pneumonia	33 (21.3)	48 (30.2)	13 (27.1)	94 (26.0)
Others ***	17 (11.4)	18 (11.3)	5 (10.4)	40 (11.0)
Referred for further care	13 (8.4)	15 (11.3)	5 (10.4)	33 (9.1)

* Normal (≥-1 for WAZ and LAZ; ≥-1 to ≤ 2 for WLZ), At risk (≥-2 to <-1), Moderate (< -2 to ≥ -3), Severe (< -3). Overweight WLZ >2 to ≤ 3, Obese WLZ > 3

** Composite malnutrition includes infants who were either underweight, stunted or wasted.

For peer review only

*** Other illnesses included abscess (1), thrush (4), scabies (8), dermatitis (3), skin infection (18), anemia (1), convulsions (3), otitis media (1), congenital cataract (1)

Neurodevelopment

Delays on one or more of the standardized neurodevelopmental assessment tools were identified in 8.6% of infants (Table 3). The 12-month-old infants were more likely to show delays than infants of the other two age groups, with gross motor and personal-social (MDAT z-score) areas most impacted. Seven children (2%) showed HINE findings indicative of cerebral palsy. In univariate analysis, a HINE score concerning for cerebral palsy was more likely in children born by C-section (OR 9.27, 95% CI 2.0-42.8) and was significantly associated with wasting (OR 2.24, 95% CI 1.05-4.80). Neurodevelopmental delay was more likely in males (OR 3.55, 95% CI 1.62-7.79) and in infants who were underweight (OR 4.01, 95% CI 1.80-8.94), stunted (OR 2.96, 95% CI 1.39-6.33), or wasted (OR 2.76, 95% CI 1.03-7.36). Overall, 22.7% of caregivers expressed some concerns on the TQQ about their child's neurodevelopment.

Table 3. Neurodevelopmental Outcomes

Age at Assessment	6 months n (%)	12 months n (%)	18 months n (%)	All n (%)
Delayed by MDAT†				
Pass/Fail criteria				
Gross Motor	6 (3.9)	9 (5.7)	0	15 (4.1)
Fine Motor	1 (0.7)	2 (1.3)	1 (2.1)	4 (1.1)
Language	0	2 (1.3)	1 (2.1)	3 (0.8)
Personal Social	1 (0.7)	2 (1.3)	1 (2.1)	4 (1.1)
Total MDAT*	8 (5.2)	12 (7.6)	3 (6.3)	23 (6.4)
<= -2 SD from Mean				
Gross Motor	0	10 (6.3)	0	10 (2.8)
Fine Motor	6 (3.9)	4 (2.5)	1 (2.1)	11 (3.0)
Language	5 (3.2)	3 (1.9)	2 (4.2)	10 (2.8)
Personal Social	3 (1.9)	15 (9.4)	0	18 (5.0)
Total MDAT*	2 (1.3)	6 (3.8)	2 (4.2)	10 (2.8)

For peer review only

Delayed by HINE†	5 (3.2)	1 (0.6)	1 (2.1)	7 (1.9)
Neurodevelopmental Delay††	12 (7.7)	15 (9.4)	4 (k8.3)	31 (8.6)
Ten Questions Questionnaire: Total with one or more concerns	18 (11.6)	43 (27.0)	21 (43.8)	82 (22.7)

† MDAT=Malawi Developmental Assessment Tool; HINE = Hammersmith Infant Neurologic Examination
†† Neurodevelopmental Delay defined as a fail on one or more of the 3 evaluation criteria, MDAT Pass/Fail, MDAT Z-score (≤ -2 standard deviations from mean) or HINE.
* NOTE: A fail score on the total MDAT can occur with a fail in any one or more subscales, thus this number does not represent the sum of children failing on the domain scores.

DISCUSSION

This study describes growth and neurodevelopmental outcomes for a rural community sample of PT/LBW survivors. Infants were similar in gestational age to other community-based samples from countries with NMR ≥ 5 and constituted a relatively low-risk sample of PT/LBW infants compared to high-resource contexts or LMIC settings with available NICU care. Only 27% were born at the county's tertiary referral hospital, with the remaining born at other rural facilities. Surviving infants would thus be expected to have better outcomes than their counterparts requiring neonatal intensive care in urban settings of Africa.

Rates of stunting, wasting and underweight were higher than locally reported data, suggesting a higher risk of malnutrition than the general population. Direct comparison to growth in the available community-based African samples is complicated by differences in country under-5 malnutrition rates when these studies took place.[13,22,23] Nonetheless, findings are concerning, particularly given low parental awareness (fewer than 3% expressed concern for acute or chronic malnutrition) and apparently limited detection/intervention at routine child health/immunization visits.

For peer review only

This study demonstrates that standardized assessments can be locally implemented to enhance neurodevelopmental evaluation at the community-level. Directly administered, standardized neurodevelopmental assessment tools identified delay or disability in 8.6% of PT/LBW infants, fewer than global estimates in settings with high NMR and NICU care available and more comparable to, but still lower than, other cited community-based studies.[3,8,10] A higher number of caregivers expressed developmental concerns, with more concern for older children, likely in part due to the increase in observable developmental milestones/skills as children age.

The HINE was successfully used as a predictive assessment for cerebral palsy or motor disability. Approximately 2% of children showed concern for being non-ambulatory by 2 years, and one additional child met clinical criteria for cerebral palsy but was not included in the sample due acute illness at time of visit. While these numbers are low, the percentage is not markedly different than the 3.4% of children with neonatal encephalopathy who had "sub-optimal" HINE scores in a recent Ugandan study.[24] With global PT births estimated at 15 million annually, even these small percentages would translate to almost 1.3 million children with developmental delay or high risk for disability annually, highlighting the importance of targeted clinical follow-up and implementation of early intervention programs for these at-risk infants in low-resource communities.[12,25]

In addition to malnutrition and neurodevelopmental risks, a high proportion of the sample were reported to have experienced acute childhood illness in their lifetime, including malaria, diarrheal disease, and serious febrile illness. Children in the current study had higher rates of acute respiratory infection in the last two weeks than local averages for under-5 children (26% vs. 13%).[13] Increased rates of respiratory and severe infections have been documented for PT

For peer review only

infants in other contexts, indicating that these major illnesses may differentially affect PT/LBW infants.[10] Although community data for the other illnesses are lacking, malaria is endemic in Migori County and a major cause of under-5 mortality (19%).[26]

Our data may underestimate true developmental delay/disability rates for PT/LBW infants for two reasons. First, participants were part of a larger cRCT evaluating the effect of an intrapartum and immediate postnatal intervention package on PT/LBW survival. Although post-hoc univariate analyses revealed no significant differences in growth or neurodevelopment between babies born at control versus intervention sites, the small sample may have masked effects to some extent, and the control arm did receive two of the four interventions.[11,12] Second, the cohort comprised largely moderate to late PT infants with predominately normal or LBW, as opposed to very or extremely PT/LBW infants, and the vast majority had 5-minute Apgar scores ≥ 7.[27] These findings are consistent with WHO data suggesting that half of babies born before 32 weeks in low-income countries will not survive; however, they suggest findings may be an underestimate of adverse outcomes of PT/LBW babies in LMIC more broadly.[28] Compared to all infants who survived to 28 days in the larger parent study, infants in this sample were more likely to be female and to have younger mothers at time of delivery. Since 79% of infants who died prior to study contact were female, survival bias is an unlikely reason for this female predominance. However, in our small cohort, males were more likely to be malnourished and have developmental delay, suggesting that additional longitudinal investigation into gender-related outcomes is warranted. Whether maternal age differences were due to differential survival or challenges in locating teen mothers is unknown; however, future research would ideally gather information on surviving PT infants among adolescent mothers in LMIC. Other

For peer review only

important sample characteristics did not differ, suggesting the sample was largely representative of the PT/LBW population.

This study has several limitations. First, the study design did not allow for direct comparison to term, normal birthweight controls, and it was not possible to investigate factors contributing to poor growth or neurodevelopmental outcomes through multivariate analyses. Additionally, there were too few babies in the highest-risk PT/LBW categories to separately investigate their neurodevelopmental outcomes. Although only 6.9% of those contacted declined to participate, 25.9% of eligible participants we attempted to contact were unreachable, and 20.4% of those scheduled for visits did not attend, suggesting possible selection bias in this subsample.

Conclusion

The current study adds to very limited community-based literature on PT/LBW infants born in countries with high NMR and suggests higher than background rates of wasting and underweight, high rates of parental concern for development, and a clinically impactful number of children with neurodevelopmental delay or risk for disability. The results highlight the need for policies that support close monitoring of and early intervention for high-risk infants to assure PT/LBW infants in both rural and urban areas of LMIC are able to thrive.

For peer review only

Acknowledgements: We wish to thank the caregivers and infants who generously gave their time to participate in this project.

Funding Source: This work was supported by the Bill & Melinda Gates Foundation under grant number OPP1107312 (East Africa Preterm Birth Initiative).

Ethics Approval: This project was approved by the Scientific and Ethics Review Unit (SERU) of the Kenya Medical Research Institute (SERU #: KEMRI/SERU/CCR/0104/3668) and the UCSF Institutional Review Board (UCSF IRB#: 18-25555). Written authorization was obtained from the Migori County Director of Health.

Data Availability: The data that support the findings of this study are available from the corresponding author, Susanne Martin-Herz, upon reasonable request.

Word Count: 3106 (4239 including Tables)

Competing interests: The authors have no financial relationships or conflicts of interest relevant to this article to disclose.

Contributorship statement: SMH, PO, GN, NS and DW contributed to study conceptualization. VM, GO led data cleaning and analysis. SMH, PO, GN, VM, GO, NS and DW contributed to data synthesis and interpretation. SMH and PO drafted and revised the manuscript, GN, VM, GO, NS and DW provided edits and feedback. All authors approved the final version of the manuscript for submission.

For peer review only

REFERENCES

1. World Health Organization. WHO Newborn Deaths and Illnesses. Published online 2011. Accessed March 20, 2018. http://www.who.int.ucsf.idm.oclc.org/pmnch/media/press_materials/fs/fs_newborndealth_illness/en/

2. Ahmed I, Ali SM, Amenga-Etego S, et al. Population-based rates, timing, and causes of maternal deaths, stillbirths, and neonatal deaths in south Asia and sub-Saharan Africa: a multi-country prospective cohort study. *The Lancet Global Health*. 2018;6(12):e1297-e1308. doi:10.1016/S2214-109X(18)30385-1

3. Blencowe H, Lee ACC, Cousens S, et al. Preterm birth-associated neurodevelopmental impairment estimates at regional and global levels for 2010. *Pediatric research*. 2013;74 Suppl 1(Journal Article):17.

4. Lawn JE, Blencowe H, Oza S, et al. Every Newborn: Progress, priorities, and potential beyond survival. *The Lancet*. 2014;384(9938):189-205.

5. Murray CJL Prof, Vos T Prof, Lozano R Prof, et al. Disability-adjusted life years (DALYs) for 291 diseases and injuries in 21 regions, 1990–2010: a systematic analysis for the Global Burden of Disease Study 2010. *Lancet, The*. 2012;380(9859):2197-2223. doi:10.1016/S0140-6736(12)61689-4

6. Gladstone M, Oliver C, Van den Broek N. Survival, morbidity, growth and developmental delay for babies born preterm in low and middle income countries - a systematic review of outcomes measured. *PloS one*. 2015;10(3):e0120566. doi:10.1371/journal.pone.0120566

7. World Health Organization. *World Health Statistics Overview 2019: Monitoring Health for the Sustainable Development Goals*. World Health Organization; 2019. Accessed July 15, 2020. https://apps.who.int/iris/bitstream/handle/10665/311696/WHO-DAD-2019.1-eng.pdf?ua=1

8. Namazzi G, Tumwine JK, Hildenwall H, et al. Neurodevelopmental outcomes of preterm babies during infancy in Eastern Uganda: a prospective cohort study. *Glob Health Action*. 2020;13(1):1820714. doi:10.1080/16549716.2020.1820714

9. Kirk CM, Uwamungu JC, Wilson K, et al. Health, nutrition, and development of children born preterm and low birth weight in rural Rwanda: a cross-sectional study. *BMC Pediatr*. 2017;17(1):191. doi:10.1186/s12887-017-0946-1

10. Gladstone M, White S, Kafulafula G, Neilson JP, van den Broek N. Post-neonatal mortality, morbidity, and developmental outcome after ultrasound-dated preterm birth in rural Malawi: a community-based cohort study. *PLoS medicine*. 2011;8(11):e1001121. doi:10.1371/journal.pmed.1001121

11. Otieno P, Waiswa P, Butrick E, et al. Strengthening intrapartum and immediate newborn care to reduce morbidity and mortality of preterm infants born in health facilities in Migori

For peer review only

County, Kenya and Busoga Region, Uganda: a study protocol for a randomized controlled trial. *Trials*. 2018;19(1):1-12. doi:10.1186/s13063-018-2696-2

12. Walker D, Otieno P, Butrick E, et al. Impact of an intrapartum and immediate newborn care quality improvement package on fresh stillbirth and neonatal mortality among preterm and low birthweight births in Kenya and Uganda: a cluster randomised facility-based trial. *Lancet Global Health*. 2020;8:e1061-70.

13. Kenya National Bureau of Statistics, DHS Program, ICF International. *Kenya 2014 Demographic and Health Survey*.; 2014. Accessed April 5, 2020. https://dhsprogram.com/pubs/pdf/fr308/fr308.pdf

14. Were FN, Bwibo NO. Neonatal nutrition and later outcomes of very low birth weight infants at Kenyatta National Hospital. *African Health Sciences*. 2007;7(2):108-114.

15. Sutton PS, Darmstadt GL. Preterm birth and neurodevelopment: a review of outcomes and recommendations for early identification and cost-effective interventions. *Journal of tropical pediatrics*. 2013;59(4):258-265. doi:10.1093/tropej/fmt012

16. Ballot DE, Potterton J, Chirwa T, Hilburn N, Cooper PA. Developmental outcome of very low birth weight infants in a developing country. *BMC pediatrics*. 2012;12(1):11. doi:10.1186/1471-2431-12-11

17. Cochran WG. *Sampling Techniques*. 3rd ed. John Wiley & Sons; 1977.

18. Gladstone M, Lancaster GA, Umar E, et al. The Malawi Developmental Assessment Tool (MDAT): the creation, validation, and reliability of a tool to assess child development in rural African settings. *PLoS medicine*. 2010;7(5):e1000273. doi:10.1371/journal.pmed.1000273

19. Frisone MF, Mercuri E, Laroche S, et al. Prognostic value of the neurologic optimality score at 9 and 18 months in preterm infants born before 31 weeks' gestation. *The Journal of Pediatrics*. 2002;140(1):57-60. doi:10.1067/mpd.2002.119626

20. World Health Organization, United Nation's Children's Fund. *WHO Child Growth Standards and the Identification of Severe Acute Malnutrition in Infants and Children. A Joint Statement*.; 2009. Accessed February 2, 2020. https://apps.who.int/nutrition/publications/severemalnutrition/9789241598163/en/index.html

21. World Health Organization. Global database on child growth. Published online 2018. Accessed April 22, 2020. https://www.who.int/nutgrowthdb/about/introduction/en/index5.html

22. National Institute of Statistics of Rwanda, Rwanda Ministry of Finance and Economic Planning, Rwanda Ministry of Health, The DHS Program, ICF International. *Rwanda Demographic and Health Survey 2014-2015*.; 2014.

For peer review only

23. National Statistics Office. *Malawi Demographic and Health Survey 2004*. National Statistics Office and ORC Macro; 2005.

24. Tann CJ, Webb EL, Lassman R, et al. Early childhood outcomes after neonatal encephalopathy in Uganda: A cohort study. *EClinicalMedicine*. 2018;6:26-35. doi:10.1016/j.eclinm.2018.12.001

25. Every Preemie Scale. *Kenya Profile of Preterm and Low Birth Weight Prevention and Care*.; 2019. Accessed August 20, 2020. https://www.everypreemie.org/wp-content/uploads/2019/07/Kenya_7.5.19.pdf

26. Starnes JR, Chamberlain L, Sutermaster S, et al. Under-five mortality in the Rongo Sub-County of Migori County, Kenya: Experience of the Lwala Community Alliance 2007-2017 with evidence from a cross-sectional survey. van Wouwe JP, ed. *PLoS ONE*. 2018;13(9):e0203690. doi:10.1371/journal.pone.0203690

27. American College of Obstetricians and Gynecologists' Task Force on Neonatal Encephalopathy. Neonatal encephalopathy and neurologic outcome, Second Edition. *PEDIATRICS*. 2014;133(5):e1482-e1488. doi:10.1542/peds.2014-0724

28. World Health Organization. *Preterm Birth*. World Health Organization; 2018. Accessed July 18, 2020. https://www.who.int/news-room/fact-sheets/detail/preterm-birth

Figure 1: Study flow diagram

For peer review only

Figure 1: Study Flow Diagram

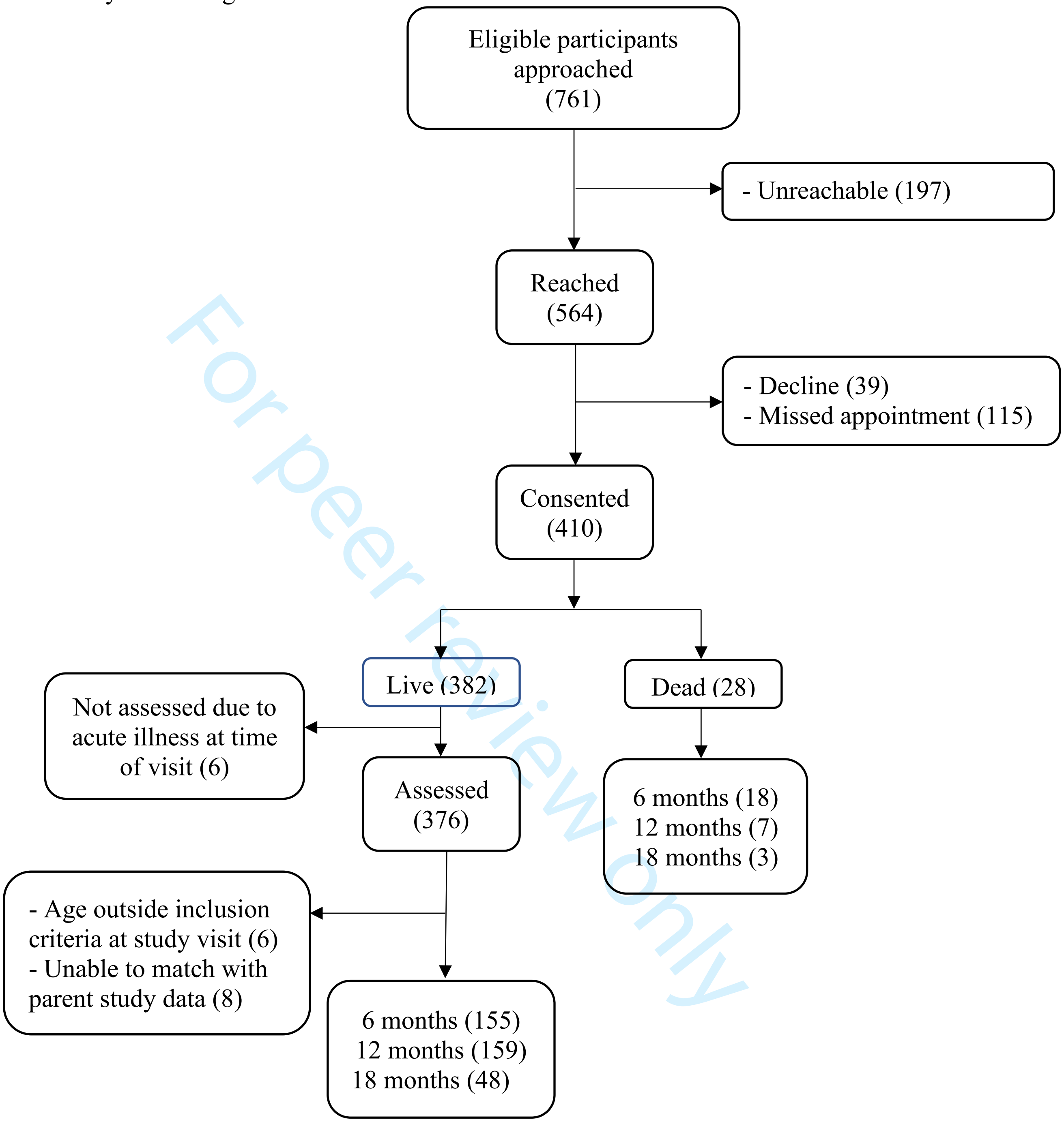

For peer review only

Supplemental Table 1. Anthropometric measurement procedures

Assessment	Methods
Weight	• Digital baby weighing scale calibrated using Standardization weight (10 kg Stone) every morning<br>• Weighing scale surface disinfected before use by the next baby<br>• Weighing scale placed on flat hard surface and made stable before placing baby on<br>• Ensured all clothing removed by caregiver (socks, diapers)<br>• Baby calmed then placed on the weighing scale (sitting or recumbent), unsupported<br>• Measurement read when the scale stopped counting, to the nearest 0.1 kg
Length	• Standard Length Mat placed on a hard flat surface with caregiver as an assistant.<br>• Length Mat surface disinfected before use by the next baby<br>• Ensured all clothing removed by caregiver (socks, diapers)<br>• Caregiver brought the child to the mat and kneeling on the left side and facing the child supported the head and neck to the correct position on mat.<br>• Assessor, kneeling on the right of child, ensured child was in perpendicular position to the base of the length mat, while supporting the knees of child, making sure the shoulders level, hands at child's side, and child's buttocks touching back of length mat<br>• Assessor moved foot piece with right hand until firmly against child's heels<br>• Measurement was read to the nearest 0.1 cm<br>• Procedure was repeated up to 2 times for confirmatory measurement
Mid upper arm circumference (MUAC)	• Used the standard measuring tape that cannot be stretched<br>• With baby on caregiver's lap, assessor exposed and positioned left arm of baby to hang loosely at the side<br>• Shoulder tip identified; tape placed at midpoint and made to run along arm<br>• With elbow flexed, tape positioned on same level, tip of elbow marked and midpoint between tip of the shoulder and tip of bent elbow identified and marked<br>• Adjusting for tension and gaps, tape placed around arm at midpoint and secured using assessor's index finger and thumb at the junction where the 0 mark of the tape meets other end of tape<br>• Measurement recorded to the nearest **0.1cm** and repeated up to 2 times for accuracy, then the average recorded
Occipital Frontal Head Circumference (OFC)	• Used a standard paper measuring tape that cannot be stretched<br>• Securely wrapped tape around widest possible circumference of the head, broadest part of forehead above eyebrow, above ears and most prominent part of back of head<br>• Measurement taken three times<br>• Largest measurement to the nearest **0.1 cm** recorded

For peer review only

Supplemental Table 2. Neurodevelopment Assessment Tools

Assessment	Description	Validity
Ten Questions Questionnaire (TQQ)[31,32]	Brief, caregiver-report screener for neurologic delay or disability. Normed for ages 2 to 9 years and adapted previously for younger children[10]<br>Delay noted if caregiver concern noted on at least one question	Acceptable sensitivity for serious disability[6,33]<br>Successfully used in African contexts[6,33]
Malawi Developmental Assessment Tool (MDAT)[10,34]	Four developmental domains: Gross motor, Fine motor & performance, Language & hearing, Social<br>Developed in Malawi as culturally relevant tool for use in Africa	Excellent reliability and good validity<br>Sensitive to differences between term and preterm infants
Hammersmith Inventory for Neurologic Examination (HINE)[35,36]	Rapid, validated, structured neurologic evaluation	High predictive validity for later cerebral palsy in children from birth to 2 years of age (90% sensitivity)<br>Successfully used in several studies in Africa, as well as clinically in Kenya

For peer review only

Supplemental Table 3. Demographic variables eligible infants from parent study versus enrolled sample for follow-up study

	Parent Study 28-day Survivors n (%)	Follow-up Study Sample n (%)
**Neonatal factors**		
Gender		
Male	1113 (47.0)	144 (39.8)
Female	1255 (53.0)	218 (60.2)
Gestational Age (weeks)		
≥ 37*	989 (36.1)	114 (31.5)
32 to <37	1131 (41.3)	205 (56.6)
28 to <32	183 (6.7)	34 (9.4)
22 to <28	29 (1.1)	6 (1.7)
Unknown	405 (14.8)	3 (0.8)
Birthweight (grams)		
2500 – 2999**	1005 (42.3)	129 (35.6)
1500 – 2499	1282 (54.0)	218 (60.2)
1000 – 1499	74 (3.1)	10 (2.8)
500 – 999	14 (0.6)	5 (1.4)
Apgar – 5 minute		
0 to 3	6 (0.2)	1 (0.3)
4 to 6	84 (3.1)	11 (3.0)

For peer review only

>= 7	2286 (83.5)	332 (91.7)
Unknown	361 (13.2)	18 (5.0)
**Maternal factors**		
Age (years)		
< 19	569 (24.0)	41 (11.3)
19 to 25	1001 (42.3)	183 (50.6)
> 25	797 (33.7)	138 (38.1)
Delivery Mode		
Vaginal	2126 (77.7)	309 (85.4)
Cesarean	230 (8.4)	48 (13.3)
Unknown	381 (13.9)	5 (13.8)

* Infants ≥ 37 weeks' gestation were included only if birthweight was < 2500 grams.

** Infants 2500 – 2999 grams were included only if gestational age was < 37 weeks.

For peer review only

STROBE 2007 (v4) Statement—Checklist of items that should be included in reports of *cross-sectional studies*

Section/Topic	Item #	Recommendation	Reported on page #
**Title and abstract**	1	(*a*) Indicate the study's design with a commonly used term in the title or the abstract	1
		(*b*) Provide in the abstract an informative and balanced summary of what was done and what was found	3
**Introduction**			
Background/rationale	2	Explain the scientific background and rationale for the investigation being reported	5-6
Objectives	3	State specific objectives, including any prespecified hypotheses	6
**Methods**			
Study design	4	Present key elements of study design early in the paper	6
Setting	5	Describe the setting, locations, and relevant dates, including periods of recruitment, exposure, follow-up, and data collection	7
Participants	6	(*a*) Give the eligibility criteria, and the sources and methods of selection of participants	7
Variables	7	Clearly define all outcomes, exposures, predictors, potential confounders, and effect modifiers. Give diagnostic criteria, if applicable	7-8
Data sources/ measurement	8*	For each variable of interest, give sources of data and details of methods of assessment (measurement). Describe comparability of assessment methods if there is more than one group	7-9
Bias	9	Describe any efforts to address potential sources of bias	7 -8
Study size	10	Explain how the study size was arrived at	7
Quantitative variables	11	Explain how quantitative variables were handled in the analyses. If applicable, describe which groupings were chosen and why	9
Statistical methods	12	(*a*) Describe all statistical methods, including those used to control for confounding	9
		(*b*) Describe any methods used to examine subgroups and interactions	9
		(*c*) Explain how missing data were addressed	10
		(*d*) If applicable, describe analytical methods taking account of sampling strategy	NA
		(*e*) Describe any sensitivity analyses	NA
**Results**			

For peer review only

Participants	13*	(a) Report numbers of individuals at each stage of study—eg numbers potentially eligible, examined for eligibility, confirmed eligible, included in the study, completing follow-up, and analysed	10
		(b) Give reasons for non-participation at each stage	10
		(c) Consider use of a flow diagram	10, Figure 1
Descriptive data	14*	(a) Give characteristics of study participants (eg demographic, clinical, social) and information on exposures and potential confounders	10-11
		(b) Indicate number of participants with missing data for each variable of interest	10, Table 1, Table 2
Outcome data	15*	Report numbers of outcome events or summary measures	11
Main results	16	(*a*) Give unadjusted estimates and, if applicable, confounder-adjusted estimates and their precision (eg, 95% confidence interval). Make clear which confounders were adjusted for and why they were included	NA
		(*b*) Report category boundaries when continuous variables were categorized	NA
		(*c*) If relevant, consider translating estimates of relative risk into absolute risk for a meaningful time period	NA
Other analyses	17	Report other analyses done—eg analyses of subgroups and interactions, and sensitivity analyses	11
**Discussion**			
Key results	18	Summarise key results with reference to study objectives	15-16
Limitations	19	Discuss limitations of the study, taking into account sources of potential bias or imprecision. Discuss both direction and magnitude of any potential bias	17-18
Interpretation	20	Give a cautious overall interpretation of results considering objectives, limitations, multiplicity of analyses, results from similar studies, and other relevant evidence	15-18
Generalisability	21	Discuss the generalisability (external validity) of the study results	17
**Other information**			
Funding	22	Give the source of funding and the role of the funders for the present study and, if applicable, for the original study on which the present article is based	19

*Give information separately for cases and controls in case-control studies and, if applicable, for exposed and unexposed groups in cohort and cross-sectional studies.

Note: An Explanation and Elaboration article discusses each checklist item and gives methodological background and published examples of transparent reporting. The STROBE checklist is best used in conjunction with this article (freely available on the Web sites of PLoS Medicine at http://www.plosmedicine.org/, Annals of Internal Medicine at http://www.annals.org/, and Epidemiology at http://www.epidem.com/). Information on the STROBE Initiative is available at www.strobe-statement.org.

For peer review only

BMJ Open

Growth and Neurodevelopmental Outcomes of Preterm and Low Birthweight Infants in Rural Kenya: a cross-sectional study

Journal:	*BMJ Open*
Manuscript ID	bmjopen-2022-064678.R1
Article Type:	Original research
Date Submitted by the Author:	28-Sep-2022
Complete List of Authors:	Martin-Herz, Susanne P. ; University of California San Francisco, Department of Pediatrics<br>Otieno, Phelgona; Kenya Medical Research Institute, Center for Clinical Research<br>Nalwa, Grace; Maseno University, Department of Paediatrics and Child Health; Jaramogi Oginga Odinga Teaching and Referral Hospital<br>Moshi, Vincent; Kenya Medical Research Institute, Center for Clinical Research<br>Olieng'o Okoth, Geofrey; Kenya Medical Research Institute, Center for Clinical Research<br>Santos, Nicole; University of California San Francisco, Institute of Global Health Sciences<br>Walker, Dilys; University of California San Fransisco, Institute for Global Health Sciences and Department of Obstetrics, Gynecology & Reproductive Sciences
**Primary Subject Heading**:	Paediatrics
Secondary Subject Heading:	Global health, Paediatrics
Keywords:	Developmental neurology & neurodisability < PAEDIATRICS, Community child health < PAEDIATRICS, NEONATOLOGY

SCHOLARONE™
Manuscripts

BMJ

I, the Submitting Author has the right to grant and does grant on behalf of all authors of the Work (as defined in the below author licence), an exclusive licence and/or a non-exclusive licence for contributions from authors who are: i) UK Crown employees; ii) where BMJ has agreed a CC-BY licence shall apply, and/or iii) in accordance with the terms applicable for US Federal Government officers or employees acting as part of their official duties; on a worldwide, perpetual, irrevocable, royalty-free basis to BMJ Publishing Group Ltd ("BMJ") its licensees and where the relevant Journal is co-owned by BMJ to the co-owners of the Journal, to publish the Work in this journal and any other BMJ products and to exploit all rights, as set out in our licence.

The Submitting Author accepts and understands that any supply made under these terms is made by BMJ to the Submitting Author unless you are acting as an employee on behalf of your employer or a postgraduate student of an affiliated institution which is paying any applicable article publishing charge ("APC") for Open Access articles. Where the Submitting Author wishes to make the Work available on an Open Access basis (and intends to pay the relevant APC), the terms of reuse of such Open Access shall be governed by a Creative Commons licence – details of these licences and which Creative Commons licence will apply to this Work are set out in our licence referred to above.

Other than as permitted in any relevant BMJ Author's Self Archiving Policies, I confirm this Work has not been accepted for publication elsewhere, is not being considered for publication elsewhere and does not duplicate material already published. I confirm all authors consent to publication of this Work and authorise the granting of this licence.

For peer review only

Growth and Neurodevelopmental Outcomes of Preterm and Low Birthweight Infants in Rural Kenya: a cross-sectional study

Susanne P. Martin-Herz, MD, PhD*
Division of Developmental Medicine, Department of Pediatrics
University of California San Francisco
Box 4054, 1825 4th Street, 6th Floor, San Francisco, CA 94143
https://orcid.org/0000-0002-2474-3904

Phelgona Otieno, MBChB, MMed Paediatrics, MPH*
Kenya Medical Research Institute, Center for Clinical Research
P.O. Box 54840 00200 Off Mbagathi Road, Nairobi, Kenya
https://orcid.org/0000-0002-2927-9848

Grace Nalwa, MBChB, MMed Paediatrics
Maseno University School of Medicine, Department of Paediatrics and Child Health
Maseno, Nyanza, Kenya

Vincent Moshi, MSc.
Kenya Medical Research Institute, Center for Clinical Research
P.O. Box 54840 00200 Off Mbagathi Road, Nairobi, Kenya

Geofrey Olieng'o Okoth, Dip. CM
Kenya Medical Research Institute, Center for Clinical Research
P.O. Box 54840 00200 Off Mbagathi Road, Nairobi, Kenya

Nicole Santos, PhD
University of California San Francisco, Institute for Global Health Sciences
550 16th St, San Francisco, CA 94158
https://orcid.org/0000-0002-7517-5177

Dilys Walker, MD
University of California San Francisco, Institute for Global Health Sciences and
Department of Obstetrics, Gynecology & Reproductive Sciences
550 16th St, San Francisco, CA 94158

* Contributed equally as co-first authors

Corresponding Author: Susanne Martin-Herz, MD, PhD, Division of Developmental Medicine at University of California San Francisco, 550 16th Street, UCSF Box 4054, San Francisco, CA 94143, 415-353-2080, Susanne.MartinHerz@ucsf.edu

Running Head: Preterm and Low Birthweight Outcome in Rural Kenya

For peer review only

Abbreviations: preterm (PT), low birthweight (LBW), Hammersmith Infant Neurological Examination (HINE), Malawi Developmental Assessment Tool (MDAT), TQQ (Ten Questions Questionnaire)

Keywords: Preterm, Low Birth Weight, Neurodevelopment

For peer review only

ABSTRACT

Objective: Data on long-term outcomes of preterm (PT) and low birthweight (LBW) infants in countries with high rates of neonatal mortality and childhood stunting are limited, especially from community settings. The current study sought to explore growth and neurodevelopmental outcomes of PT/LBW infants from a rural community-based setting of Kenya up to 18 months adjusted age.

Design: Cross-sectional study.

Setting: Migori County, Kenya.

Participants: Four hundred ten PT/LBW infants were recruited from a cluster randomized control trial evaluating a package of facility-based quality of care interventions around the time of birth.

Outcome measures: Caregiver interviews and infant health, growth and neurodevelopmental assessments were completed at 6, 12 or 18 months $\pm$ 2 weeks. Data included sociodemographic information, medical history, growth measurements, and neurodevelopmental assessment using the Ten Questions Questionnaire, Malawi Developmental Assessment Tool, and Hammersmith Infant Neurological Examination. Analyses were descriptive. No alterations were made to planned data collection.

Results: The final sample included 362 PT/LBW infants, of which 56.6% were moderate to late PT infants and 64% were LBW. Fewer than 2% of parents identified their child as malnourished, but direct measurement revealed higher proportions of stunting and underweight than in national demographic and health survey reports. Overall, 22.7% of caregivers expressed concern about their child's neurodevelopmental status. Neurodevelopmental delays were identified in 8.6% of infants based on one or more standardized tools, and 2% showed neurologic findings indicative of cerebral palsy.

Conclusions: Malnutrition and neurodevelopmental delays are common among PT/LBW infants in this setting. Close monitoring and access to early intervention programs are needed in order to help these vulnerable infants thrive.

Trial registration: Participants were recruited from an existing cluster randomized control trial (NCT03112018); however, no randomization or related analyses were conducted in the presented cross-sectional study.

Strengths and Limitations:

- This study utilized directly administered, standardized neurodevelopmental assessment tools to enhance evaluation at the community-level.
- The sample included largely moderate to late preterm (PT) infants, with predominately normal or low birthweight (LBW), as opposed to very or extremely PT/LBW infants and, therefore, may underestimate true rates of neurodevelopmental delays or disability.
- The study design did not allow direct comparison to term, appropriate birthweight controls.
- It was not possible to investigate factors contributing to poor growth or neurodevelopmental outcomes through multivariate analyses due to sample size constraints.

For peer review only

INTRODUCTION:

Complications associated with preterm (PT) birth and low birthweight (LBW) contribute to 25% to 50% of all neonatal deaths and 12% of under-5 mortality worldwide.[1,2] Additionally, close to one million PT survivors experience neurodevelopmental impairments each year, and PT birth is the fifth leading cause of Disability Adjusted Life Years (DALYs) in East Africa.[3–5] However, there is a paucity of data on the long-term outcomes of both PT and LBW infants in countries with high neonatal mortality rates (NMR), particularly from community settings.[6] In countries with an NMR ≥ 5, global estimates suggest approximately 24.6% of PT survivors are at risk of moderate or severe neurodevelopmental impairment and 32.5% of mild neurodevelopmental disability; however, these estimates are based on only 7 datasets, all in settings with neonatal intensive units (NICU).[3]

Data from community-based PT/LBW samples in areas without NICUs are extremely limited, meaning outcomes of the majority of PT/LBW infants born in low-income countries (LMIC) are not represented in current estimates.[6,7] Three community-based, rural cohort studies from Malawi, Rwanda and Uganda exist, showing PT or LBW babies to be significantly more likely than term infants to have died between 6 weeks and 24 months adjusted age, with death rates twice as high for premature infants at 1 and 2 years than for term infants.[8–10] Survivors were more commonly wasted or underweight.[8,10] Additionally, caregivers of PT infants were significantly more likely to express concern about their child's development than caregivers of term infants; up to two-thirds of PT/LBW infants in the Rwandan sample showed developmental delays on a standardized, validated caregiver-report developmental screening tool at an average age of 22.5 months.[9,10] PT survivors were also significantly more likely to have neurodevelopmental delays on directly-administered assessments than term counterparts, with

particular deficits in the language and fine motor domains. Being underweight or malnourished was significantly associated with delays for both term and PT infants.[8,10]

In Kenya, an estimated 12% and 10.5% of births are PT and LBW, respectively.[11,12] In Migori County, where the current study took place, rates of malnutrition in children under-5 include stunting in 26.4%, underweight in 8.6%, and wasting in 4%.[13] One study from a Kenyan urban, academic center followed very LBW (VLBW, <1500g) infants for 2 years post-discharge and found 11.7% (95% CI, 6.2-17.1) had cerebral palsy, 9.2% (95% Cl 4.2-16.9) had cognitive delay, and 26.7% (95% Cl, 12.2-36.9) had functional disability.[14] However, this sample is likely not representative of rural sites that lack NICU services.

Early interventions (e.g., physio-, occupational and speech therapies, family support and training) increasingly show improvements in long-term outcomes of PT and other at-risk babies, both in high-income settings and LMIC, highlighting the need for additional studies to better understand growth and neurodevelopment of PT/LBW infants across community settings.[6,15,16] The current study leveraged the Preterm Birth Initiative Kenya (PTBi-K) cohort[11] to explore growth and neurodevelopment of PT/LBW infants up to 18 months adjusted age in Migori County, Kenya and provides data towards better understanding of health and neurodevelopmental outcomes among PT/LBW infants at the rural, community level.

MATERIALS AND METHODS:

Design. This cross-sectional study was conducted between October 2018 to May 2019 among a subset of mothers and babies previously enrolled in PTBi-K, a cluster randomized control trial (cRCT) of a package of interventions to improve quality of care during labor and the immediate postnatal period (Clinical Trials Registration: NCT03112018). The protocol and primary results

of this cRCT have been published elsewhere.[11,12] The current cross-sectional study was not designed to evaluate the impact of the cRCT intervention package.

Setting. The current study was conducted in Migori County, Kenya. The county is mostly rural, has poor access to health care and has higher infant and under-5 mortality than national statistics (50 vs. 39 per 1000 live births, and 82 vs. 52 per 1000 live births, respectively).[13]

Study Participants and Sampling Strategy. Participants in the parent cRCT were identified from maternity registers. Eligible participants were LBW (<2500g at birth) or PT (gestational age <37 weeks with birthweight <3000g) infants delivered at one of 17 facilities across the county. A list of potentially eligible infants, alive at 28 days and approaching 6, 12 or 18 months ± 2 weeks of age was created, with age adjusted for preterm status if the infant was born at less than 37 weeks' gestation. Recruitment was sequential toward the goal sample size.

A priori calculation of sample size using the Cochran's method was based on the caregiver-report Ten Questions Questionnaire (TQQ) in a community-based study of PT versus term infants in Malawi.[10,17] The calculated target sample size was n=183 per age group to detect a delay prevalence of 0.139 with a power of at least 80% and median effect size of 0.3.

Procedures. Caregivers of eligible infants were contacted via phone, and a standard participation invitation script was used to explain the study. Appointments were scheduled at a study facility nearest the family's home. All consent forms and questionnaires were translated and back translated from English to Kiswahili and Dholuo.

Pregnancy, birth and neonatal course data were extracted from the cRCT database and confirmed with the caregiver when possible. Assessors were blind to the child's birthweight and gestational age, and questions regarding these variables were not asked at the study visit. The

For peer review only

sequence of assessments was: (1) caregiver interview for sociodemographic information, medical history including growth, illness, and development, and the TQQ; (2) direct neurodevelopmental assessments including the Malawi Developmental Assessment Tool (MDAT) and Hammersmith Infant Neurological Examination (HINE); and (3) physical examination including anthropometric measurements. Details of the anthropometric measurement standardized guidelines and the 3 neurodevelopmental assessment tools are in Supplemental Tables 1 and 2.

All assessments were conducted in a conducive environment, when the child was settled and in relatively stable health, and complied with health and safety procedures. The research team consisted of clinical officers and nurses, all trained in study procedures and certified to conduct neurodevelopmental assessments. Two team members were present for each assessment, with one conducting the assessment and one observing and recording findings. A pediatrician trained in all study procedures provided consultation and regular supervision.

After assessment, feedback on the child's neurodevelopment and health was given to the caregiver and their concerns addressed. Caregivers were also given information on nutrition, danger signs for common childhood illnesses, and simple games to play with their child. Children identified with any significant health or developmental concern, such as hearing impairment, acute malnutrition or neurodevelopmental delay, were referred to appropriate follow-up care customized to the identified need (e.g., audiology, nutrition support), with costs of up to 4 care visits covered by the study.

Data collection was paper based, with subsequent entry into a Microsoft Access database. Double entry and verification to test for logical sequence, discrepancies and outliers was completed. Data were de-identified and stored on an encrypted server within a locked study facility. Efforts to address potential bias included sequential recruitment toward sample size goal,

For peer review only

reporting of differences between consenting individuals and the eligible sample, similar procedures at multiple sites to reduce loss to follow-up risk that might be associated with travel to a central location, and blinding of assessors as to child's birthweight and gestational age.

Patient and public involvement. For the larger parent study in which participants were involved, national and community advisory boards provided input on intervention priorities. Health facility providers, managers, and local authorities were involved in implementation activities and influenced the focus and content of those activities on the basis of their roles and priorities.

While caregiver participants were not involved in the design or conduct of this cross-sectional study, other than being a participant, findings specific to their child's data were disseminated directly to caregivers at the visit. If health conditions or neurodevelopmental delays were identified, clinical referrals were made as well.

Ethical considerations. This project was approved by the Scientific and Ethics Review Unit of the Kenya Medical Research Institute (KEMRI/SERU/CCR/0104/3668) and the University of California San Francisco Institutional Review Board (UCSF IRB#: 18-25555). Written authorization was obtained from the Migori County Director of Health. Formal written informed consent procedures were completed in the preferred language of each caregiver.

Statistical Analysis. Analyses were primarily descriptive and completed using STATA Version 13.0 Stata/MP. Child medical experiences were summarized as past medical illnesses (since birth) or current medical status (within 2 weeks of the assessment). MDAT and HINE total and domain scores were calculated. MDAT scores were investigated using 2 methods. First, a child was noted to have failed the MDAT overall if they were unable to complete 2 or more items in any one domain that would be expected to be passed by 90% of the normal reference population at their age.[18] Second, developmental z-scores were calculated using the most current MDAT

For peer review only

Scoring Application (beta test version v1.1), and scores were dichotomized as either typical (> -2 standard deviations (SD) of mean) or delayed (≤ -2 SD to mean). For the HINE, a score of < 64 was used, as this has been shown to be 98% predictive of walking at 2 years with a sensitivity of 85% for PT children.[19] TQQ findings were described per age group, with overall caregiver concern noted if one or more items were endorsed.

For growth, World Health Organization (WHO) child growth standards were used in calculation of z-scores as provided in the STATA igrowup package.[20] Nutritional status z-scores of weight for age (WAZ), length for age (LAZ) and weight for length (WLZ) were calculated.[21] Outcomes were categorized into normal (≥-1 for WAZ and LAZ; ≥-1 to ≤ 2 for WLZ), at risk (≥-2 to <-1), moderate (< -2 to ≥ -3) or severe (< -3). Overweight and obese were defined as WLZ >2 to ≤ 3 and WLZ > 3, respectively. A composite dichotomous malnutrition variable was created with those meeting moderate or severe criteria in at least one of the three nutritional z-score variables considered malnourished.

All available data were included in the analyses. There were few missing datapoints, and any cases of missingness for pregnancy, infant and child health characteristics are noted in Tables 1 and 2. No datapoints were missing for the MDAT or the HINE. One 12-month-old did not have a complete TQQ. Records with missing data were omitted only for each respective analysis.

RESULTS

Of 761 eligible infants, 410 (54%) consented. A total of 28 infants (7.2%) died prior to study contact, six were not assessed due to acute illness at the time of appointment, and 14 were excluded due to data mismatch. The final sample consisted of a total 362 infants (88%) with viable data, of which 155, 159 and 48 were 6, 12 and 18 months of age respectively (Figure 1).

For peer review only

The target sample size of 193 per age group was not reached due to the parent study ending earlier than expected and a national health worker strike that particularly restricted the pool of eligible 18-month-olds.

Characteristics at Delivery and Immediate Postnatal Period

Most babies were female (60.2%) and moderate to late PT (56.6%, >32 weeks' gestation; median gestational age and range = 36.3 weeks (22.0, 41.7). Of infants born preterm, 66.1% were late preterm (34 to <37 weeks), 17.6% were moderate preterm (32 to <34 weeks), 13.9% were very preterm (28 to <32), and only 2.5% were extremely preterm (22 to <28). Birthweight was over 2500g for 35.6%, and more than 90% had 5-minute Apgar scores ≥7. Sixteen percent were admitted to the newborn unit, and 35.6% needed special care (i.e., oxygen, phototherapy, kangaroo mother care) in the first month of life. Approximately 50.6% of mothers were aged 19 to 25. Most were multiparous (70.4%), and 13% of deliveries were by C-section (Table 1).

Table 1: Delivery and Immediate Postnatal Period Characteristics

Age at Assessment	6 months n (%)	12 months n (%)	18 months n (%)	All n (%)
**Neonatal factors**				
Gender				
Male	57 (36.8)	64 (40.3)	23 (47.9)	144 (39.8)
Female	98 (63.2)	95 (59.8)	25 (52.1)	218 (60.2)
Multiple pregnancy (Twins)	50 (32.3)	46 (28.9)	10 (20.8)	106 (29.3)
Gestational Age (weeks)				
≥ 37*	59 (38.1)	45 (28.3)	10 (20.8)	114 (31.5)
34 to <37	60 (38.7)	78 (49.1)	24 (50.0)	162 (44.8)
32 to <34	16 (10.3)	18 (11.3)	9 (18.8)	43 (11.9)
28 to <32	17 (11.0)	12 (7.6)	5 (10.4)	34 (9.4)
22 to <28	3 (1.9)	3 (1.9)	0	6 (1.7)
Unknown	0	3 (1.9)	0	3 (0.8)
Birthweight (grams)				
2500 – 2999**	50 (32.3)	58 (36.5)	21 (43.8)	129 (35.6)
1500 – 2499	94 (60.7)	97 (61.0)	27 (56.2)	218 (60.2)
1000 – 1499	7 (4.5)	3 (1.9)	0	10 (2.8)
500 – 999	4 (2.6)	1 (0.6)	0	5 (1.4)

For peer review only

Apgar – 5-minute				
0 to 3	0	0	1 (2.1)	1 (0.3)
4 to 6	7 (4.5)	4 (2.5)	0	11 (3.0)
>= 7	141 (91.0)	144 (90.6)	47 (97.9)	332 (91.7)
Unknown	7 (4.5)	11 (6.9)	0	18 (5.0)
Admitted to Newborn Unit (Yes)	28 (18.1)	22 (13.8)	8 (16.7)	58 (16.0)
"Special care" in first month (Yes)	59 (38.0)	59 (37.1)	11 (22.9)	129 (35.6)
Oxygen	19 (32.2)	8 (13.6)	3 (27.3)	30 (23.3)
Phototherapy	3 (5.1)	4 (6.8)	0 (0)	7 (5.4)
Kangaroo Mother Care	52 (88.1)	56 (89.8)	10 (90.9)	118 (91.5)
**Maternal factors**				
Age (years)				
< 19	24 (15.5)	12 (7.6)	5 (10.4)	41 (11.3)
19 to 25	71 (45.8)	85 (53.5)	27 (56.3)	183 (50.6)
> 25	60 (38.7)	62 (39.0)	16 (33.3)	138 (38.1)
Parity				
Primigravida	51 (32.9)	46 (28.9)	10 (20.8)	107 (29.6)
Multigravida	104 (67.1)	113 (71.1)	38 (79.2)	255 (70.4)
Delivery Mode				
Vaginal	125 (80.7)	144 (90.6)	40 (83.3)	309 (85.4)
Cesarean	26 (16.8)	14 (8.8)	8 (16.7)	48 (13.3)
Unknown	4 (2.6)	1 (0.1)		5 (13.8)

* Infants ≥ 37 weeks' gestation were included only if birthweight was < 2500 grams.
** Infants 2500 – 2999 grams were included only if gestational age was < 37 weeks.

Compared to the eligible pool of caregivers and infants from the parent study, mothers in the current study were older on average (24.7 years vs. 23.6 years, t=3.16, p<0.005), and babies were more likely female (60.2% vs. 52.8%, χ^2=7.73, p=0.02). The two groups did not differ significantly in other key demographic variables (Supplemental Table 3).

Growth and Health

Anthropometric measurement and caregiver-reported health findings are in Table 2. The prevalence of stunting, underweight, and wasting in the study population were 27.4%, 17.2% and 3.3%, respectively. The proportions of children with malnutrition increased with infant age. Moderate to severe malnutrition was significantly more common in males than females (OR

For peer review only

2.53, 95% CI 1.62-3.97), and in babies born after multiple gestation (OR 1.72, 95% CI 1.08-2.75) or with birthweight 1500 to 2499g (OR 1.73, 95% CI 1.07-2.81).

The most common illnesses reported as ever experienced by participants included malaria (56.7%), diarrheal disease (55.2%) serious febrile illness (42.3%); and in the past 2 weeks prior to assessment, respiratory tract infections (26%).

Table 2. Child Characteristics at Time of Visit

Age at Assessment	6 months n (%)	12 months n (%)	18 months n (%)	All n (%)
Weight for Age Z-score (WAZ; Underweight; valid n=343) *				
Normal	87 (58.4)	68 (45.3)	27 (61.4)	182 (53.1)
At risk	43 (28.9)	52 (34.7)	7 (15.9)	102 (29.7)
Moderate	13 (8.7)	20 (13.3)	7 (15.9)	40 (11.7)
Severe	6 (4.0)	10 (6.7)	3 (6.8)	19 (5.5)
Length for Age Z-score (LAZ; Stunting; valid n=351) *				
Normal	71 (46.7)	62 (40.5)	20 (43.5)	153 (43.6)
At risk	47 (30.9)	47 (30.7)	8 (17.4)	102 (29.1)
Moderate	24 (15.8)	26 (17.0)	11 (23.9)	61 (17.4)
Severe	10 (6.6)	18 (11.8)	7 (15.2)	35 (10.0)
Weight for Length Z-score (WLZ; Wasting; valid n=339)*				
Normal	107 (73.3)	89 (59.7)	31 (70.5)	227 (67.0)
At risk	22 (15.1)	38 (25.5)	7 (15.9)	67 (19.8)
Moderate	5 (3.4)	11 (7.4)	4 (9.1)	20 (5.9)
Severe	5 (3.4)	7 (4.7)	2 (4.6)	14 (4.1)
Overweight	6 (4.1)	4 (2.7)	0	10 (3.0)
Obese	1 (0.7)	0	0	1 (0.3)
Composite Malnutrition (Underweight/Stunted/Wasting)**				
Normal	111 (71.6)	100 (62.9)	26 (54.2)	237 (65.5)
Malnourished	44 (28.4)	59 (37.1)	20 (41.7)	123 (34.0)
Missing	0	0	2 (4.2)	2 (0.6)
Past Medical Illnesses (birth until study evaluation)				
Pneumonia	9 (5.8)	13 (8.2)	6 (12.5)	28 (7.7)
Diarrheal Disease	63 (40.7)	107 (67.3)	30 (62.5)	200 (55.2)
Seizures	8 (5.2)	22 (13.8)	4 (8.3)	34 (9.4)
Malaria	55 (35.5)	107 (67.3)	43 (89.6)	205 (56.7)
Serious febrile illness/ meningitis	28 (58.3)	41 (26.5)	84 (52.8)	153 (42.3)
Cough for > 2 weeks	13 (8.4)	26 (16.4)	5 (10.4)	44 (12.2)
Malnutrition	2 (1.3)	3 (1.9)	3 (6.2)	8 (2.2)

For peer review only

Skin infections	26 (16.8)	51 (32.1)	15 (31.3)	92 (25.4)
Current Medical Illness (in past 2 weeks)				
Acute febrile illness	3 (1.9)	1 (0.6)	0	4 (1.1)
Gastroenteritis/dysentery	21 (13.5)	20 (12.6)	6 (12.5)	47 (13.0)
Acute Malnutrition	2 (1.3)	2 (1.3)	0	4 (1.1)
Respiratory tract infection/ pneumonia	33 (21.3)	48 (30.2)	13 (27.1)	94 (26.0)
Others ***	17 (11.4)	18 (11.3)	5 (10.4)	40 (11.0)
Referred for further care	13 (8.4)	15 (11.3)	5 (10.4)	33 (9.1)

* Normal (≥-1 for WAZ and LAZ; ≥-1 to ≤ 2 for WLZ), At risk (≥-2 to <-1), Moderate (< -2 to ≥ -3), Severe (< -3). Overweight WLZ >2 to ≤ 3, Obese WLZ > 3
** Composite malnutrition includes infants who were either underweight, stunted or wasted.
*** Other illnesses included abscess (1), thrush (4), scabies (8), dermatitis (3), skin infection (18), anemia (1), convulsions (3), otitis media (1), congenital cataract (1)

Neurodevelopment

Delays on one or more of the standardized neurodevelopmental assessment tools were identified in 8.6% of infants (Table 3). The 12-month-old infants were more likely to show delays than infants of the other two age groups, with gross motor and personal-social (MDAT z-score) areas most impacted. Seven children (2%) showed HINE findings indicative of cerebral palsy. In univariate analysis, a HINE score concerning for cerebral palsy was more likely in children born by C-section (OR 9.27, 95% CI 2.0-42.8) and was significantly associated with wasting (OR 2.24, 95% CI 1.05-4.80). Neurodevelopmental delay was more likely in males (OR 3.55, 95% CI 1.62-7.79) and in infants who were underweight (OR 4.01, 95% CI 1.80-8.94), stunted (OR 2.96, 95% CI 1.39-6.33), or wasted (OR 2.76, 95% CI 1.03-7.36). Overall, 22.7% of caregivers expressed some concern about their child's neurodevelopment on the TQQ.

Table 3. Neurodevelopmental Outcomes

Age at Assessment	6 months n (%)	12 months n (%)	18 months n (%)	All n (%)
Delayed by MDAT†				
Pass/Fail criteria				
Gross Motor	6 (3.9)	9 (5.7)	0	15 (4.1)

For peer review only

Fine Motor	1 (0.7)	2 (1.3)	1 (2.1)	4 (1.1)
Language	0	2 (1.3)	1 (2.1)	3 (0.8)
Personal Social	1 (0.7)	2 (1.3)	1 (2.1)	4 (1.1)
Total MDAT*	8 (5.2)	12 (7.6)	3 (6.3)	23 (6.4)
<= -2 SD from Mean				
Gross Motor	0	10 (6.3)	0	10 (2.8)
Fine Motor	6 (3.9)	4 (2.5)	1 (2.1)	11 (3.0)
Language	5 (3.2)	3 (1.9)	2 (4.2)	10 (2.8)
Personal Social	3 (1.9)	15 (9.4)	0	18 (5.0)
Total MDAT*	2 (1.3)	6 (3.8)	2 (4.2)	10 (2.8)
Delayed by HINE†	5 (3.2)	1 (0.6)	1 (2.1)	7 (1.9)
Neurodevelopmental Delay††	12 (7.7)	15 (9.4)	4 (k8.3)	31 (8.6)
Ten Questions Questionnaire:				
Total with one or more concerns	18 (11.6)	43 (27.0)	21 (43.8)	82 (22.7)

† MDAT=Malawi Developmental Assessment Tool; HINE = Hammersmith Infant Neurologic Examination
†† Neurodevelopmental Delay defined as a fail on one or more of the 3 evaluation criteria, MDAT Pass/Fail, MDAT Z-score (≤ -2 standard deviations from mean) or HINE.
* NOTE: A fail score on the total MDAT can occur with a fail in any one or more subscales, thus this number does not represent the sum of children failing on the domain scores.

As described previously, this study recruited infants who had participated in a cRCT. The number of infants with neurological delay were small in both control and intervention groups, and the sample was not large enough to be adequately powered to detect significant group differences if present. These data are provided for review in Supplemental Tables 4 and 5, but should be interpreted with caution.

DISCUSSION

This study describes growth and neurodevelopmental outcomes for a rural community sample of PT/LBW survivors. Infants were similar in gestational age to other community-based samples from countries with NMR ≥ 5 and constituted a relatively low-risk sample of PT/LBW

For peer review only

infants compared to high-resource contexts or LMIC settings with available NICU care. Only 27% were born at the county's tertiary referral hospital, with the remaining born at other rural facilities. Surviving infants would thus be expected to have better outcomes than their counterparts requiring neonatal intensive care in urban settings of Africa.

Rates of stunting and underweight were higher than locally reported data, suggesting a higher risk of malnutrition in the current PT/LBW sample than in general population of young children in the local community. Direct comparison to growth data from available community-based African samples is complicated by differences in country under-5 malnutrition rates when these studies took place.[13,22,23] Nonetheless, findings are concerning, particularly given low parental awareness (fewer than 3% expressed concern for acute or chronic malnutrition) and apparently limited detection/intervention at routine child health/immunization visits. These findings suggest that future work focused on caregiver understanding of appropriate growth in infants born PT or LBW will be important to assuring early detection and management.

This study demonstrates that standardized assessments can be locally implemented to enhance neurodevelopmental evaluation at the community level. Directly administered, standardized neurodevelopmental assessment tools identified delay or disability in 8.6% of PT/LBW infants. This proportion is lower than global estimates from settings with high NMR but NICU care available, where one might anticipate higher risk infants surviving. It is more comparable to, but still lower than that of other cited community-based studies.[3,8,10] A higher number of caregivers expressed developmental concerns, with more concern for older children, likely in part due to the increase in observable developmental milestones/skills as children age.

The HINE was successfully used as a predictive assessment for cerebral palsy or motor disability. Approximately 2% of children showed concern for being non-ambulatory by 2 years,

For peer review only

and one additional child met clinical criteria for cerebral palsy but was not included in the sample due acute illness at the time of visit. While these numbers are low, the percentage is not markedly different than the 3.4% of children with neonatal encephalopathy who had “sub-optimal” HINE scores in a recent Ugandan study.[24] With global PT births estimated at 15 million annually, even these small percentages would translate to almost 1.3 million children with developmental delay or high risk for disability annually, highlighting the importance of targeted clinical follow-up and implementation of early intervention programs for these at-risk infants in low-resource communities.[12,25]

In addition to malnutrition and neurodevelopmental risks, a high proportion of the sample were reported to have experienced acute childhood illness in their lifetime, including malaria, diarrheal disease, and serious febrile illness. Children in the current study had higher rates of acute respiratory infection in the last two weeks than local averages for children under 5 years (26% vs. 13%).[13] Increased rates of respiratory and severe infections have been documented for PT infants in other contexts, indicating that these major illnesses may differentially affect PT/LBW infants.[10] Although community data for the other illnesses are lacking, malaria is endemic in Migori County and a major cause of under-5 mortality (19%).[26]

Our data may underestimate true developmental delay/disability rates for PT/LBW infants for two reasons. First, participants were part of a larger cRCT evaluating the effect of an intrapartum and immediate postnatal intervention package on PT/LBW neonatal survival in which the control arm also received two of the four interventions. Post-hoc univariate analyses revealed no significant differences in growth or neurodevelopment between babies born at control versus intervention sites (Supplemental Tables 4 and 5); however, these findings should be considered with caution due to the small sample size and because this cross-sectional study

For peer review only

was not designed to evaluate the impact of the cRCT intervention on growth or neurodevelopmental outcomes.[11,12] Second, study participants were largely moderate to late PT infants with predominately normal or LBW, as opposed to very or extremely PT/LBW infants, and the vast majority had 5-minute Apgar scores ≥ 7.[27] These findings are consistent with WHO data suggesting that half of babies born before 32 weeks in low-income countries will not survive; however, they suggest findings may be an underestimate of adverse outcomes of PT/LBW babies in LMIC more broadly.[28] Compared to all infants who survived to 28 days in the larger parent study, infants in this sample were more likely to be female and to have younger mothers at time of delivery (Supplemental Table 3). Since 79% of infants who died prior to study contact were female, survival bias is an unlikely reason for this female predominance. However, in our small sample, males were more likely to be malnourished and have developmental delay, suggesting that additional longitudinal investigation into gender-related outcomes is warranted. Whether maternal age differences were due to differential survival or challenges in locating teen mothers is unknown; however, future research would ideally gather information on surviving PT infants among adolescent mothers in LMIC. Other important sample characteristics did not differ, suggesting the sample was largely representative of the PT/LBW population. In contrast, there is the possibility that these data may bias somewhat toward higher risk of health, growth, and neurodevelopmental difficulties, since almost 30% of participating infants were born after twin pregnancies. Future studies with long-term follow-up of PT/LBW infants may consider including only singleton births or planning a priori for additional analyses comparing infants from singleton pregnancies with those born after twin pregnancies.

This study has several limitations. First, the study design did not allow for direct comparison to term, normal birthweight controls, and it was not possible to investigate factors

For peer review only

contributing to poor growth or neurodevelopmental outcomes through multivariate analyses. Additionally, there were too few babies in the highest-risk PT/LBW categories to separately investigate their neurodevelopmental outcomes. Although only 6.9% of those contacted declined to participate, 25.9% of eligible participants we attempted to contact were unreachable, and 20.4% of those scheduled for visits did not attend, suggesting possible selection bias in this subsample.

Conclusion

The current study adds to very limited community-based literature on PT/LBW infants born in countries with high NMR and suggests higher than background rates of wasting and underweight, high rates of parental concern for development, and a clinically impactful number of children with neurodevelopmental delay or risk for disability. The results highlight the need for policies that support close monitoring of and early intervention for high-risk infants to assure PT/LBW infants in both rural and urban areas of LMIC are able to thrive.

For peer review only

Acknowledgements: We wish to thank the caregivers and infants who generously gave their time to participate in this project.

Funding Source: This work was supported by the Bill & Melinda Gates Foundation under grant number OPP1107312 (East Africa Preterm Birth Initiative).

Ethics Approval: This project was approved by the Scientific and Ethics Review Unit (SERU) of the Kenya Medical Research Institute (SERU #: KEMRI/SERU/CCR/0104/3668) and the UCSF Institutional Review Board (UCSF IRB#: 18-25555). Written authorization was obtained from the Migori County Director of Health.

Data Availability: The data that support the findings of this study are available from the corresponding author, Susanne Martin-Herz, upon reasonable request.

Word Count: 3106 (4239 including Tables)

Competing interests: The authors have no financial relationships or conflicts of interest relevant to this article to disclose.

Contributorship statement: SMH, PO, GN, NS and DW contributed to study conceptualization. VM, GO led data cleaning and analysis. SMH, PO, GN, VM, GO, NS and DW contributed to data synthesis and interpretation. SMH and PO drafted and revised the manuscript, GN, VM, GO, NS and DW provided edits and feedback. All authors approved the final version of the manuscript for submission.

For peer review only

REFERENCES

1. World Health Organization. WHO Newborn Deaths and Illnesses. Published online 2011. Accessed March 20, 2018. http://www.who.int.ucsf.idm.oclc.org/pmnch/media/press_materials/fs/fs_newborndealth_illness/en/

2. Ahmed I, Ali SM, Amenga-Etego S, et al. Population-based rates, timing, and causes of maternal deaths, stillbirths, and neonatal deaths in south Asia and sub-Saharan Africa: a multi-country prospective cohort study. *The Lancet Global Health*. 2018;6(12):e1297-e1308. doi:10.1016/S2214-109X(18)30385-1

3. Blencowe H, Lee ACC, Cousens S, et al. Preterm birth-associated neurodevelopmental impairment estimates at regional and global levels for 2010. *Pediatric research*. 2013;74 Suppl 1(Journal Article):17.

4. Lawn JE, Blencowe H, Oza S, et al. Every Newborn: Progress, priorities, and potential beyond survival. *The Lancet*. 2014;384(9938):189-205.

5. Murray CJL Prof, Vos T Prof, Lozano R Prof, et al. Disability-adjusted life years (DALYs) for 291 diseases and injuries in 21 regions, 1990–2010: a systematic analysis for the Global Burden of Disease Study 2010. *Lancet, The*. 2012;380(9859):2197-2223. doi:10.1016/S0140-6736(12)61689-4

6. Gladstone M, Oliver C, Van den Broek N. Survival, morbidity, growth and developmental delay for babies born preterm in low and middle income countries - a systematic review of outcomes measured. *PloS one*. 2015;10(3):e0120566. doi:10.1371/journal.pone.0120566

7. World Health Organization. *World Health Statistics Overview 2019: Monitoring Health for the Sustainable Development Goals*. World Health Organization; 2019. Accessed July 15, 2020. https://apps.who.int/iris/bitstream/handle/10665/311696/WHO-DAD-2019.1-eng.pdf?ua=1

8. Namazzi G, Tumwine JK, Hildenwall H, et al. Neurodevelopmental outcomes of preterm babies during infancy in Eastern Uganda: a prospective cohort study. *Glob Health Action*. 2020;13(1):1820714. doi:10.1080/16549716.2020.1820714

9. Kirk CM, Uwamungu JC, Wilson K, et al. Health, nutrition, and development of children born preterm and low birth weight in rural Rwanda: a cross-sectional study. *BMC Pediatr*. 2017;17(1):191. doi:10.1186/s12887-017-0946-1

10. Gladstone M, White S, Kafulafula G, Neilson JP, van den Broek N. Post-neonatal mortality, morbidity, and developmental outcome after ultrasound-dated preterm birth in rural Malawi: a community-based cohort study. *PLoS medicine*. 2011;8(11):e1001121. doi:10.1371/journal.pmed.1001121

11. Otieno P, Waiswa P, Butrick E, et al. Strengthening intrapartum and immediate newborn care to reduce morbidity and mortality of preterm infants born in health facilities in Migori

For peer review only

County, Kenya and Busoga Region, Uganda: a study protocol for a randomized controlled trial. *Trials*. 2018;19(1):1-12. doi:10.1186/s13063-018-2696-2

12. Walker D, Otieno P, Butrick E, et al. Impact of an intrapartum and immediate newborn care quality improvement package on fresh stillbirth and neonatal mortality among preterm and low birthweight births in Kenya and Uganda: a cluster randomised facility-based trial. *Lancet Global Health*. 2020;8:e1061-70.

13. Kenya National Bureau of Statistics, DHS Program, ICF International. *Kenya 2014 Demographic and Health Survey*.; 2014. Accessed April 5, 2020. https://dhsprogram.com/pubs/pdf/fr308/fr308.pdf

14. Were FN, Bwibo NO. Neonatal nutrition and later outcomes of very low birth weight infants at Kenyatta National Hospital. *African Health Sciences*. 2007;7(2):108-114.

15. Sutton PS, Darmstadt GL. Preterm birth and neurodevelopment: a review of outcomes and recommendations for early identification and cost-effective interventions. *Journal of tropical pediatrics*. 2013;59(4):258-265. doi:10.1093/tropej/fmt012

16. Ballot DE, Potterton J, Chirwa T, Hilburn N, Cooper PA. Developmental outcome of very low birth weight infants in a developing country. *BMC pediatrics*. 2012;12(1):11. doi:10.1186/1471-2431-12-11

17. Cochran WG. *Sampling Techniques*. 3rd ed. John Wiley & Sons; 1977.

18. Gladstone M, Lancaster GA, Umar E, et al. The Malawi Developmental Assessment Tool (MDAT): the creation, validation, and reliability of a tool to assess child development in rural African settings. *PLoS medicine*. 2010;7(5):e1000273. doi:10.1371/journal.pmed.1000273

19. Frisone MF, Mercuri E, Laroche S, et al. Prognostic value of the neurologic optimality score at 9 and 18 months in preterm infants born before 31 weeks' gestation. *The Journal of Pediatrics*. 2002;140(1):57-60. doi:10.1067/mpd.2002.119626

20. World Health Organization, United Nation's Children's Fund. *WHO Child Growth Standards and the Identification of Severe Acute Malnutrition in Infants and Children. A Joint Statement*.; 2009. Accessed February 2, 2020. https://apps.who.int/nutrition/publications/severemalnutrition/9789241598163/en/index.html

21. World Health Organization. Global database on child growth. Published online 2018. Accessed April 22, 2020. https://www.who.int/nutgrowthdb/about/introduction/en/index5.html

22. National Institute of Statistics of Rwanda, Rwanda Ministry of Finance and Economic Planning, Rwanda Ministry of Health, The DHS Program, ICF International. *Rwanda Demographic and Health Survey 2014-2015*.; 2014.

For peer review only

23. National Statistics Office. *Malawi Demographic and Health Survey 2004*. National Statistics Office and ORC Macro; 2005.

24. Tann CJ, Webb EL, Lassman R, et al. Early childhood outcomes after neonatal encephalopathy in Uganda: A cohort study. *EClinicalMedicine*. 2018;6:26-35. doi:10.1016/j.eclinm.2018.12.001

25. Every Preemie Scale. *Kenya Profile of Preterm and Low Birth Weight Prevention and Care*.; 2019. Accessed August 20, 2020. https://www.everypreemie.org/wp-content/uploads/2019/07/Kenya_7.5.19.pdf

26. Starnes JR, Chamberlain L, Sutermaster S, et al. Under-five mortality in the Rongo Sub-County of Migori County, Kenya: Experience of the Lwala Community Alliance 2007-2017 with evidence from a cross-sectional survey. van Wouwe JP, ed. *PLoS ONE*. 2018;13(9):e0203690. doi:10.1371/journal.pone.0203690

27. American College of Obstetricians and Gynecologists' Task Force on Neonatal Encephalopathy. Neonatal encephalopathy and neurologic outcome, Second Edition. *PEDIATRICS*. 2014;133(5):e1482-e1488. doi:10.1542/peds.2014-0724

28. World Health Organization. *Preterm Birth*. World Health Organization; 2018. Accessed July 18, 2020. https://www.who.int/news-room/fact-sheets/detail/preterm-birth

Figure 1: Study flow diagram

For peer review only

Figure 1: Study Flow Diagram

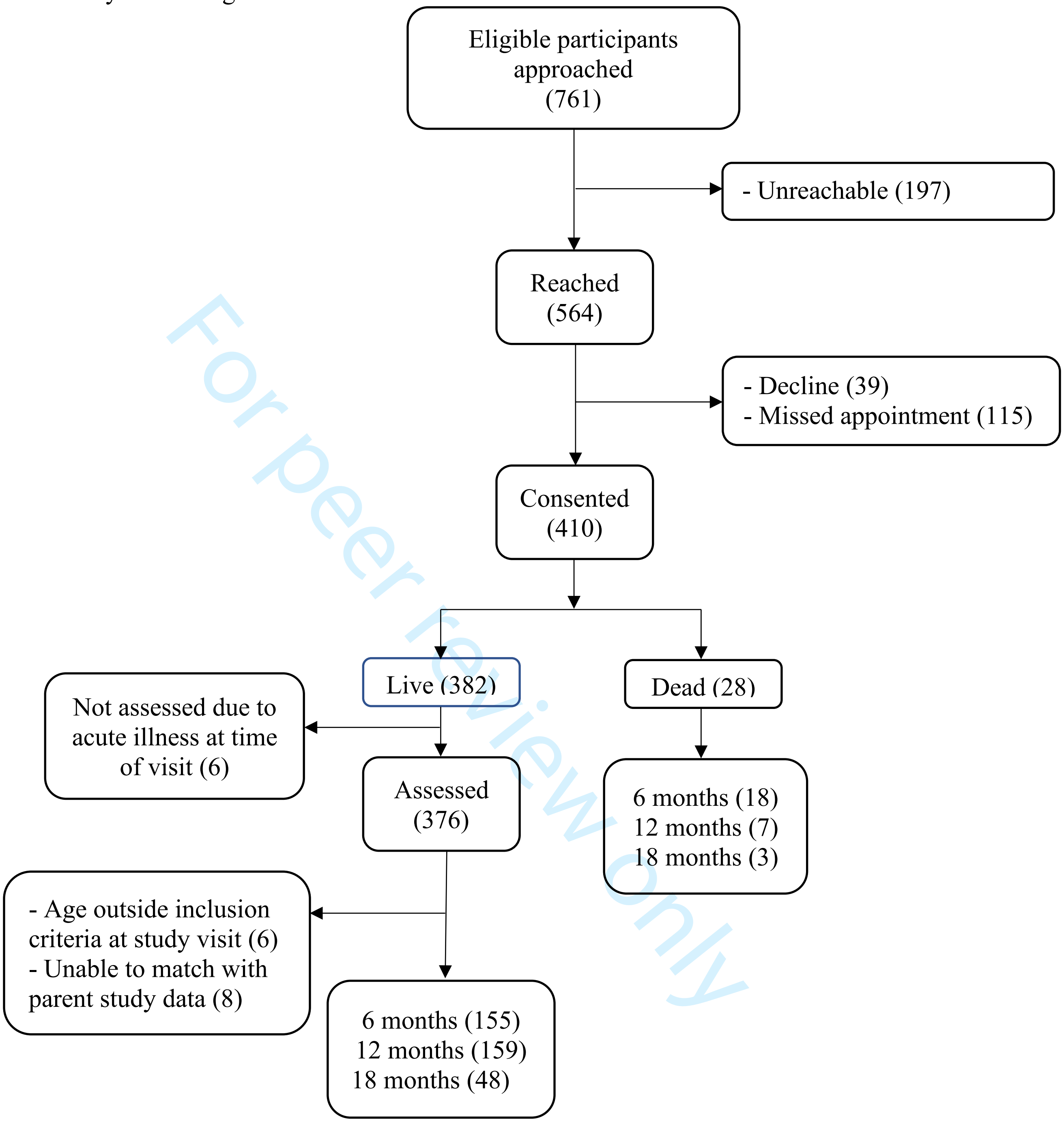

For peer review only

Supplemental Table 1. Anthropometric measurement procedures

Assessment	Methods
Weight	• Digital baby weighing scale calibrated using Standardization weight (10 kg Stone) every morning<br>• Weighing scale surface disinfected before use by the next baby<br>• Weighing scale placed on flat hard surface and made stable before placing baby on<br>• Ensured all clothing removed by caregiver (socks, diapers)<br>• Baby calmed then placed on the weighing scale (sitting or recumbent), unsupported<br>• Measurement read when the scale stopped counting, to the nearest 0.1 kg
Length	• Standard Length Mat placed on a hard flat surface with caregiver as an assistant.<br>• Length Mat surface disinfected before use by the next baby<br>• Ensured all clothing removed by caregiver (socks, diapers)<br>• Caregiver brought the child to the mat and kneeling on the left side and facing the child supported the head and neck to the correct position on mat.<br>• Assessor, kneeling on the right of child, ensured child was in perpendicular position to the base of the length mat, while supporting the knees of child, making sure the shoulders level, hands at child's side, and child's buttocks touching back of length mat<br>• Assessor moved foot piece with right hand until firmly against child's heels<br>• Measurement was read to the nearest 0.1 cm<br>• Procedure was repeated up to 2 times for confirmatory measurement
Mid upper arm circumference (MUAC)	• Used the standard measuring tape that cannot be stretched<br>• With baby on caregiver's lap, assessor exposed and positioned left arm of baby to hang loosely at the side<br>• Shoulder tip identified; tape placed at midpoint and made to run along arm<br>• With elbow flexed, tape positioned on same level, tip of elbow marked and midpoint between tip of the shoulder and tip of bent elbow identified and marked<br>• Adjusting for tension and gaps, tape placed around arm at midpoint and secured using assessor's index finger and thumb at the junction where the 0 mark of the tape meets other end of tape<br>• Measurement recorded to the nearest **0.1cm** and repeated up to 2 times for accuracy, then the average recorded
Occipital Frontal Head Circumference (OFC)	• Used a standard paper measuring tape that cannot be stretched<br>• Securely wrapped tape around widest possible circumference of the head, broadest part of forehead above eyebrow, above ears and most prominent part of back of head<br>• Measurement taken three times<br>• Largest measurement to the nearest **0.1 cm** recorded

For peer review only

Supplemental Table 2. Neurodevelopment Assessment Tools

Assessment	Description	Validity
Ten Questions Questionnaire (TQQ)[31,32]	Brief, caregiver-report screener for neurologic delay or disability. Normed for ages 2 to 9 years and adapted previously for younger children[10]<br>Delay noted if caregiver concern noted on at least one question	Acceptable sensitivity for serious disability[6,33]<br>Successfully used in African contexts[6,33]
Malawi Developmental Assessment Tool (MDAT)[10,34]	Four developmental domains: Gross motor, Fine motor & performance, Language & hearing, Social<br>Developed in Malawi as culturally relevant tool for use in Africa	Excellent reliability and good validity<br>Sensitive to differences between term and preterm infants
Hammersmith Inventory for Neurologic Examination (HINE)[35,36]	Rapid, validated, structured neurologic evaluation	High predictive validity for later cerebral palsy in children from birth to 2 years of age (90% sensitivity)<br>Successfully used in several studies in Africa, as well as clinically in Kenya

For peer review only

Supplemental Table 3. Demographic variables eligible infants from parent study versus enrolled sample for follow-up study

	Parent Study (cRCT) 28-day Survivors n (%)	Follow-up Study Sample n (%)
**Neonatal factors**		
Gender		
Male	1113 (47.0)	144 (39.8)
Female	1255 (53.0)	218 (60.2)
Gestational Age (weeks)		
≥ 37*	989 (36.1)	114 (31.5)
32 to <37	1131 (41.3)	205 (56.6)
28 to <32	183 (6.7)	34 (9.4)
22 to <28	29 (1.1)	6 (1.7)
Unknown	405 (14.8)	3 (0.8)
Birthweight (grams)		
2500 – 2999**	1005 (42.3)	129 (35.6)
1500 – 2499	1282 (54.0)	218 (60.2)
1000 – 1499	74 (3.1)	10 (2.8)
500 – 999	14 (0.6)	5 (1.4)
Apgar – 5 minute		
0 to 3	6 (0.2)	1 (0.3)
4 to 6	84 (3.1)	11 (3.0)
>= 7	2286 (83.5)	332 (91.7)
Unknown	361 (13.2)	18 (5.0)
**Maternal factors**		
Age (years)		
< 19	569 (24.0)	41 (11.3)
19 to 25	1001 (42.3)	183 (50.6)
> 25	797 (33.7)	138 (38.1)
Delivery Mode		
Vaginal	2126 (77.7)	309 (85.4)
Cesarean	230 (8.4)	48 (13.3)
Unknown	381 (13.9)	5 (13.8)

* Infants ≥ 37 weeks' gestation were included only if birthweight was < 2500 grams.
** Infants 2500 – 2999 grams were included only if gestational age was < 37 weeks.
cRTC = cluster randomized control trial

For peer review only

Supplemental Table 4. Child Characteristics by Parent Study (cRCT) Arm

	Intervention n (%)	Control n (%)	All n (%)
Weight for Age Z-score (WAZ; Underweight; valid n=343) *			
Normal	121 (51.9)	61 (55.5)	182 (53.1)
At risk	71 (30.5)	31 (28.2)	102 (29.7)
Moderate	28 (12.0)	12 (10.9)	40 (11.7)
Severe	13 (5.6)	6 (5.5)	19 (5.5)
Length for Age Z-score (LAZ; Stunting; valid n=351) *			
Normal	100 (42.2)	53 (46.5)	153 (43.6)
At risk	81 (34.2)	21 (18.4)	102 (29.1)
Moderate	34 (14.4)	27 (23.7)	61 (17.4)
Severe	22 (9.3)	13 (11.4)	35 (10.0)
Weight for Length Z-score (WLZ; Wasting; valid n=339)*			
Normal	152 (67.3)	75 (66.4)	227 (67.0)
At risk	40 (17.7)	27 (23.9)	67 (19.8)
Moderate	15 (6.6)	5 (4.4)	20 (5.9)
Severe	11 (4.9)	3 (2.7)	14 (4.1)
Overweight	7 (3.1)	3 (2.7)	10 (3.0)
Obese	1 (0.4)	0	1 (0.3)
Composite Malnutrition (Underweight/Stunted/Wasting)**			
Normal	163 (66.5)	74 (63.3)	237 (65.5)
Malnourished	80 (32.7)	43 (36.8)	123 (34.0)
Missing	2 (0.8)	0	2 (0.6)
Past Medical Illnesses (birth until study evaluation)			
Pneumonia	18 (7.4)	10 (8.6)	28 (7.7)
Diarrheal Disease	121 (49.4)	71 (60.7)	192 (53.0)
Seizures	24 (9.8)	10 (8.6)	34 (9.4)
Malaria	141 (57.6)	64 (54.7)	205 (56.6)
Serious febrile illness/meningitis	97 (39.6)	55 (47.0)	152 (42.0)
Cough for > 2 weeks	33 (13.5)	11 (9.4)	44 (12.2)
Malnutrition	5 (2.0)	3 (2.6)	8 (2.2)
Skin infections	60 (24.5)	32 (27.4)	92 (25.4)
Current Medical Illness (in past 2 weeks)			
Acute febrile illness	3 (1.2)	1 (0.9)	4 (1.1)
Gastroenteritis/dysentery	33 (13.5)	14 (12.0)	47 (13.0)
Acute Malnutrition	4 (1.6)	0	4 (1.1)
Respiratory tract infection/pneumonia	76 (31.0)	18 (15.4)	94 (26.0)
Others ***	30 (12.2)	12 (10.3)	42 (11.6)
Referred for further care	27 (11.0)	8 (6.8)	35 (9.7)

For peer review only

* Normal (≥-1 for WAZ and LAZ; ≥-1 to ≤ 2 for WLZ), At risk (≥-2 to <-1), Moderate (< -2 to ≥ -3), Severe (< -3). Overweight WLZ >2 to ≤ 3, Obese WLZ > 3
** Composite malnutrition includes infants who were either underweight, stunted or wasted.
*** Other illnesses included acute conjunctivitis (1), abscess (1), thrush (4), scabies (8), dermatitis (3), skin infection (18), anemia (1), convulsions (3), otitis media (1), congenital cataract (1), worm infection (1)
cRTC = cluster randomized control trial
Note: The current study was not designed to assess the impact of the intervention on these variables. These data are presented for information only.

For peer review only

Supplemental Table 5. Neurodevelopmental Outcomes by Parent Study (cRCT) Arm

	Intervention n (%)	Control n (%)	All n (%)
Delayed by MDAT†			
Pass/Fail criteria			
Gross Motor	11 (4.5)	4 (3.4)	15 (4.1)
Fine Motor	2 (0.8)	2 (1.7)	4 (1.1)
Language	0	3 (2.6)	3 (0.8)
Personal Social	3 (1.2)	1 (0.9)	4 (1.1)
Total MDAT*	14 (5.7)	9 (7.7)	23 (6.4)
<= -2 SD from Mean			
Gross Motor	8 (3.3)	2 (1.7)	10 (2.8)
Fine Motor	8 (3.3)	3 (2.6)	11 (3.0)
Language	6 (2.5)	4 (3.4)	10 (2.8)
Personal Social	13 (5.3)	5 (4.3)	18 (5.0)
Total MDAT*	7 (2.9)	3 (2.6)	10 (2.8)
Delayed by HINE†	6 (2.5)	1 (0.9)	7 (1.9)
Neurodevelopmental Delay††	20 (8.2)	11 (9.4)	31 (8.6)
Ten Questions Questionnaire:			
Total with one or more concerns	61 (24.9)	21 (18.0)	82 (22.7)

† MDAT=Malawi Developmental Assessment Tool; HINE = Hammersmith Infant Neurologic Examination
†† Neurodevelopmental Delay defined as a fail on one or more of the 3 evaluation criteria, MDAT Pass/Fail, MDAT Z-score (≤ -2 standard deviations from mean) or HINE.
* NOTE: A fail score on the total MDAT can occur with a fail in any one or more subscales, thus this number does not represent the sum of children failing on the domain scores.
cRTC = cluster randomized control trial
Note: The current study was not designed to assess the impact of the intervention on these variables. These data are presented for information only.

For peer review only

STROBE 2007 (v4) Statement—Checklist of items that should be included in reports of *cross-sectional studies*

Section/Topic	Item #	Recommendation	Reported on page #
**Title and abstract**	1	(*a*) Indicate the study's design with a commonly used term in the title or the abstract	1
		(*b*) Provide in the abstract an informative and balanced summary of what was done and what was found	3
**Introduction**			
Background/rationale	2	Explain the scientific background and rationale for the investigation being reported	5-6
Objectives	3	State specific objectives, including any prespecified hypotheses	6
**Methods**			
Study design	4	Present key elements of study design early in the paper	6
Setting	5	Describe the setting, locations, and relevant dates, including periods of recruitment, exposure, follow-up, and data collection	7
Participants	6	(*a*) Give the eligibility criteria, and the sources and methods of selection of participants	7
Variables	7	Clearly define all outcomes, exposures, predictors, potential confounders, and effect modifiers. Give diagnostic criteria, if applicable	7-8
Data sources/ measurement	8*	For each variable of interest, give sources of data and details of methods of assessment (measurement). Describe comparability of assessment methods if there is more than one group	7-9
Bias	9	Describe any efforts to address potential sources of bias	7 -8
Study size	10	Explain how the study size was arrived at	7
Quantitative variables	11	Explain how quantitative variables were handled in the analyses. If applicable, describe which groupings were chosen and why	9
Statistical methods	12	(*a*) Describe all statistical methods, including those used to control for confounding	9
		(*b*) Describe any methods used to examine subgroups and interactions	9
		(*c*) Explain how missing data were addressed	10
		(*d*) If applicable, describe analytical methods taking account of sampling strategy	NA
		(*e*) Describe any sensitivity analyses	NA
**Results**			

For peer review only

Participants	13*	(a) Report numbers of individuals at each stage of study—eg numbers potentially eligible, examined for eligibility, confirmed eligible, included in the study, completing follow-up, and analysed	10
		(b) Give reasons for non-participation at each stage	10
		(c) Consider use of a flow diagram	10, Figure 1
Descriptive data	14*	(a) Give characteristics of study participants (eg demographic, clinical, social) and information on exposures and potential confounders	10-11
		(b) Indicate number of participants with missing data for each variable of interest	10, Table 1, Table 2
Outcome data	15*	Report numbers of outcome events or summary measures	11
Main results	16	(*a*) Give unadjusted estimates and, if applicable, confounder-adjusted estimates and their precision (eg, 95% confidence interval). Make clear which confounders were adjusted for and why they were included	NA
		(*b*) Report category boundaries when continuous variables were categorized	NA
		(*c*) If relevant, consider translating estimates of relative risk into absolute risk for a meaningful time period	NA
Other analyses	17	Report other analyses done—eg analyses of subgroups and interactions, and sensitivity analyses	11
**Discussion**			
Key results	18	Summarise key results with reference to study objectives	15-16
Limitations	19	Discuss limitations of the study, taking into account sources of potential bias or imprecision. Discuss both direction and magnitude of any potential bias	17-18
Interpretation	20	Give a cautious overall interpretation of results considering objectives, limitations, multiplicity of analyses, results from similar studies, and other relevant evidence	15-18
Generalisability	21	Discuss the generalisability (external validity) of the study results	17
**Other information**			
Funding	22	Give the source of funding and the role of the funders for the present study and, if applicable, for the original study on which the present article is based	19

*Give information separately for cases and controls in case-control studies and, if applicable, for exposed and unexposed groups in cohort and cross-sectional studies.

Note: An Explanation and Elaboration article discusses each checklist item and gives methodological background and published examples of transparent reporting. The STROBE checklist is best used in conjunction with this article (freely available on the Web sites of PLoS Medicine at http://www.plosmedicine.org/, Annals of Internal Medicine at http://www.annals.org/, and Epidemiology at http://www.epidem.com/). Information on the STROBE Initiative is available at www.strobe-statement.org.

For peer review only

BMJ Open

Growth and Neurodevelopmental Outcomes of Preterm and Low Birthweight Infants in Rural Kenya: a Cross-sectional Study

Journal:	*BMJ Open*
Manuscript ID	bmjopen-2022-064678.R2
Article Type:	Original research
Date Submitted by the Author:	31-Mar-2023
Complete List of Authors:	Martin-Herz, Susanne P. ; University of California San Francisco, Department of Pediatrics<br>Otieno, Phelgona; Kenya Medical Research Institute, Center for Clinical Research<br>Nalwa, Grace; Maseno University, Department of Paediatrics and Child Health; Jaramogi Oginga Odinga Teaching and Referral Hospital<br>Moshi, Vincent; Kenya Medical Research Institute, Center for Clinical Research<br>Olieng'o Okoth, Geofrey; Kenya Medical Research Institute, Center for Clinical Research<br>Santos, Nicole; University of California San Francisco, Institute of Global Health Sciences<br>Walker, Dilys; University of California San Fransisco, Institute for Global Health Sciences and Department of Obstetrics, Gynecology & Reproductive Sciences
**Primary Subject Heading**:	Paediatrics
Secondary Subject Heading:	Global health
Keywords:	Developmental neurology & neurodisability < PAEDIATRICS, Community child health < PAEDIATRICS, NEONATOLOGY

SCHOLARONE™ Manuscripts

For peer review only - http://bmjopen.bmj.com/site/about/guidelines.xhtml

I, the Submitting Author has the right to grant and does grant on behalf of all authors of the Work (as defined in the below author licence), an exclusive licence and/or a non-exclusive licence for contributions from authors who are: i) UK Crown employees; ii) where BMJ has agreed a CC-BY licence shall apply, and/or iii) in accordance with the terms applicable for US Federal Government officers or employees acting as part of their official duties; on a worldwide, perpetual, irrevocable, royalty-free basis to BMJ Publishing Group Ltd ("BMJ") its licensees and where the relevant Journal is co-owned by BMJ to the co-owners of the Journal, to publish the Work in this journal and any other BMJ products and to exploit all rights, as set out in our licence.

The Submitting Author accepts and understands that any supply made under these terms is made by BMJ to the Submitting Author unless you are acting as an employee on behalf of your employer or a postgraduate student of an affiliated institution which is paying any applicable article publishing charge ("APC") for Open Access articles. Where the Submitting Author wishes to make the Work available on an Open Access basis (and intends to pay the relevant APC), the terms of reuse of such Open Access shall be governed by a Creative Commons licence – details of these licences and which Creative Commons licence will apply to this Work are set out in our licence referred to above.

Other than as permitted in any relevant BMJ Author's Self Archiving Policies, I confirm this Work has not been accepted for publication elsewhere, is not being considered for publication elsewhere and does not duplicate material already published. I confirm all authors consent to publication of this Work and authorise the granting of this licence.

For peer review only

Growth and Neurodevelopmental Outcomes of Preterm and Low Birthweight Infants in Rural Kenya: a Cross-sectional Study

Susanne P. Martin-Herz, MD, PhD*
Division of Developmental Medicine, Department of Pediatrics
University of California San Francisco
Box 4054, 1825 4th Street, 6th Floor, San Francisco, CA 94143
https://orcid.org/0000-0002-2474-3904

Phelgona Otieno, MBChB, MMed Paediatrics, MPH*
Kenya Medical Research Institute, Center for Clinical Research
P.O. Box 54840 00200 Off Mbagathi Road, Nairobi, Kenya
https://orcid.org/0000-0002-2927-9848

Grace Nalwa, MBChB, MMed Paediatrics
Maseno University School of Medicine, Department of Paediatrics and Child Health
Maseno, Nyanza, Kenya

Vincent Moshi, MSc.
Kenya Medical Research Institute, Center for Clinical Research
P.O. Box 54840 00200 Off Mbagathi Road, Nairobi, Kenya

Geofrey Olieng'o Okoth, Dip. CM
Kenya Medical Research Institute, Center for Clinical Research
P.O. Box 54840 00200 Off Mbagathi Road, Nairobi, Kenya

Nicole Santos, PhD
University of California San Francisco, Institute for Global Health Sciences
550 16th St, San Francisco, CA 94158
https://orcid.org/0000-0002-7517-5177

Dilys Walker, MD
University of California San Francisco, Institute for Global Health Sciences and
Department of Obstetrics, Gynecology & Reproductive Sciences
550 16th St, San Francisco, CA 94158

* Contributed equally as co-first authors

Corresponding Author: Susanne Martin-Herz, MD, PhD, Division of Developmental Medicine at University of California San Francisco, 550 16th Street, UCSF Box 4054, San Francisco, CA 94143, 415-353-2080, Susanne.MartinHerz@ucsf.edu

Running Head: Preterm and Low Birthweight Outcome in Rural Kenya

For peer review only

Abbreviations: preterm (PT), low birthweight (LBW), Hammersmith Infant Neurological Examination (HINE), Malawi Developmental Assessment Tool (MDAT), TQQ (Ten Questions Questionnaire)

Keywords: Preterm, Low Birth Weight, Neurodevelopment

For peer review only

ABSTRACT

Objective: Data on long-term outcomes of preterm (PT) and low birthweight (LBW) infants in countries with high rates of neonatal mortality and childhood stunting are limited, especially from community settings. The current study sought to explore growth and neurodevelopmental outcomes of PT/LBW infants from a rural community-based setting of Kenya up to 18 months adjusted age.

Design: Cross-sectional study.

Setting: Migori County, Kenya.

Participants: Three hundred eighty-two PT/LBW infants (50.2% of those identified as eligible) from a cluster randomized control trial evaluating a package of facility-based intrapartum quality of care interventions for newborn survival consented for follow-up.

Outcome measures: Caregiver interviews and infant health, growth and neurodevelopmental assessments were completed at 6, 12 or 18 months $\pm$ 2 weeks. Data included sociodemographic information, medical history, growth measurements, and neurodevelopmental assessment using the Ten Questions Questionnaire, Malawi Developmental Assessment Tool, and Hammersmith Infant Neurological Examination. Analyses were descriptive and univariate regression models. No alterations were made to planned data collection.

Results: The final sample included 362 PT/LBW infants, of which 56.6% were moderate to late PT infants and 64.4% were LBW. Fewer than 2% of parents identified their child as currently malnourished, but direct measurement revealed higher proportions of stunting and underweight than in national demographic and health survey reports. Overall, 22.7% of caregivers expressed concern about their child's neurodevelopmental status. Neurodevelopmental delays were identified in 8.6% of infants based on one or more standardized tools, and 1.9% showed neurologic findings indicative of cerebral palsy.

Conclusions: Malnutrition and neurodevelopmental delays are common among PT/LBW infants in this setting. Close monitoring and access to early intervention programs are needed to help these vulnerable infants thrive.

Trial registration: Participants were recruited from an existing cluster randomized control trial (NCT03112018); however, no randomization or related analyses were conducted in the presented cross-sectional study.

Strengths and Limitations:

- This study utilized directly administered, standardized neurodevelopmental assessment tools to enhance evaluation at the community-level.
- The sample included largely moderate to late preterm (PT) infants, with predominately normal or low birthweight (LBW), as opposed to very or extremely PT/LBW infants and, therefore, may underestimate true rates of neurodevelopmental delays or disability.
- The study design did not allow direct comparison to term, appropriate birthweight controls.

For peer review only

- It was not possible to investigate factors contributing to poor growth or neurodevelopmental outcomes through multivariate analyses due to sample size constraints.

For peer review only

INTRODUCTION:

Complications associated with preterm (PT) birth and low birthweight (LBW) contribute to 25% to 50% of all neonatal deaths and 12% of under-5 mortality worldwide.(1,2) Additionally, close to one million PT survivors experience neurodevelopmental impairments each year, and PT birth is the fifth leading cause of Disability Adjusted Life Years (DALYs) in East Africa.(3–5) However, there is a paucity of data on the long-term outcomes of both PT and LBW infants in countries with high neonatal mortality rates (NMR), particularly from community settings.(6) In countries with an NMR ≥ 5, global estimates suggest approximately 24.6% of PT survivors are at risk of moderate or severe neurodevelopmental impairment and 32.5% of mild neurodevelopmental disability; however, these estimates are based on only 7 datasets, all in settings with neonatal intensive units (NICU).(3)

Data from community-based PT/LBW samples in areas without NICUs are extremely limited, meaning outcomes of the majority of PT/LBW infants born in low-income countries (LMIC) are not represented in current estimates.(6,7) Three community-based, rural cohort studies from Malawi, Rwanda and Uganda exist, showing PT or LBW babies to be significantly more likely than term infants to have died between 6 weeks and 24 months adjusted age, with death rates twice as high for premature infants at 1 and 2 years than for term infants.(8–10) Survivors were more commonly wasted or underweight.(8,10) Additionally, caregivers of PT infants were significantly more likely to express concern about their child’s development than caregivers of term infants; up to two-thirds of PT/LBW infants in the Rwandan sample showed developmental delays on a standardized, validated caregiver-report developmental screening tool at an average age of 22.5 months.(9,10) PT survivors were also significantly more likely to have neurodevelopmental delays on directly-administered assessments than term counterparts, with

particular deficits in the language and fine motor domains. Being underweight or malnourished was significantly associated with delays for both term and PT infants.(8,10)

In Kenya, an estimated 12% and 10.5% of births are PT and LBW, respectively.(11,12) In Migori County, where the current study took place, rates of malnutrition in children under-5 include stunting in 26.4%, underweight in 8.6%, and wasting in 4%.(13) One study from a Kenyan urban, academic center followed very LBW (VLBW, <1500g) infants for 2 years post-discharge and found 11.7% (95% CI, 6.2-17.1) had cerebral palsy, 9.2% (95% Cl 4.2-16.9) had cognitive delay, and 26.7% (95% Cl, 12.2-36.9) had functional disability.(14) However, this sample is likely not representative of rural sites that lack NICU services.

Early interventions (e.g., physio-, occupational and speech therapies, family support and training) increasingly show improvements in long-term outcomes of PT and other at-risk babies, both in high-income settings and LMIC, highlighting the need for additional studies to better understand growth and neurodevelopment of PT/LBW infants across community settings.(6,15,16) The current study leveraged the Preterm Birth Initiative Kenya (PTBi-K) cohort(11) to explore growth and neurodevelopment of PT/LBW infants up to 18 months adjusted age in Migori County, Kenya and provides data towards better understanding of health and neurodevelopmental outcomes among PT/LBW infants at the rural, community level.

MATERIALS AND METHODS:

Design. This cross-sectional study was conducted between October 2018 to May 2019 among a subset of mothers and babies previously enrolled in PTBi-K, a cluster randomized control trial (cRCT) of a package of interventions to improve quality of care during labor and the immediate postnatal period and evaluate the intervention's impact on stillbirth and neonatal survival (Clinical Trials Registration: NCT03112018). The protocol and primary results of this cRCT

have been published elsewhere.(11,12) The current cross-sectional study was not designed to evaluate the impact of the cRCT intervention package.

Setting. The current study was conducted in Migori County, Kenya. The county is mostly rural, has poor access to health care and has higher infant and under-5 mortality than national statistics (50 vs. 39 per 1000 live births, and 82 vs. 52 per 1000 live births, respectively).(13)

Study Participants and Sampling Strategy. Participants in the parent cRCT were identified from maternity registers. Eligible participants were LBW (<2500g at birth) or PT (gestational age <37 weeks with birthweight <3000g) infants delivered at one of 17 facilities across the county. A list of potentially eligible infants, alive at 28 days and approaching 6, 12 or 18 months ± 2 weeks of age was created, with age adjusted for preterm status if the infant was born at less than 37 weeks' gestation. Recruitment was sequential toward the goal sample size across combined cRCT arms, as this follow-up study was not designed to evaluate the impact of the cRCT intervention.

A priori calculation of sample size using the Cochran's method was based on the caregiver-report Ten Questions Questionnaire (TQQ) in a community-based study of PT versus term infants in Malawi.(10,17) The calculated target sample size was n=183 per age group to detect a delay prevalence of 0.139 with a power of at least 80% and precision of 0.05.

Procedures. Caregivers of eligible infants were contacted via phone, and a standard participation invitation script was used to explain the study. Appointments were scheduled at a study facility nearest the family's home. All consent forms and questionnaires were translated and back translated from English to Kiswahili and Dholuo.

Pregnancy, birth and neonatal course data were extracted from the cRCT database and confirmed with the caregiver when possible. Assessors were blind to the child's birthweight and

For peer review only

gestational age, and questions regarding these variables were not asked at the study visit. The sequence of assessments was: (1) caregiver interview for sociodemographic information, medical history including growth, illness, and development, and the TQQ; (2) direct neurodevelopmental assessments including the Malawi Developmental Assessment Tool (MDAT) and Hammersmith Infant Neurological Examination (HINE); and (3) physical examination including anthropometric measurements. Details of the anthropometric measurement standardized guidelines and the 3 neurodevelopmental assessment tools are in Supplemental Tables 1 and 2.

All assessments were conducted in a conducive environment, when the child was settled and in relatively stable health, and complied with health and safety procedures. The research team consisted of clinical officers and nurses, all trained in study procedures and certified to conduct neurodevelopmental assessments. Two team members were present for each assessment, with one conducting the assessment and one observing and recording findings. A pediatrician trained in all study procedures provided consultation and regular supervision.

After assessment, feedback on the child's neurodevelopment and health was given to the caregiver and their concerns addressed. Caregivers were also given information on nutrition, danger signs for common childhood illnesses, and simple games to play with their child. Children identified with any significant health or developmental concern, such as hearing impairment, acute malnutrition or neurodevelopmental delay, were referred to appropriate follow-up care customized to the identified need (e.g., audiology, nutrition support), with costs of up to 4 care visits covered by the study.

Data collection was paper based, with subsequent entry into a Microsoft Access database. Double entry and verification to test for logical sequence, discrepancies and outliers was completed. Data were de-identified and stored on an encrypted server within a locked study

For peer review only

facility. Efforts to address potential bias included sequential recruitment toward sample size goal, reporting of differences between consenting individuals and the eligible sample, similar procedures at multiple sites to reduce loss to follow-up risk that might be associated with travel to a central location, and blinding of assessors as to child's birthweight and gestational age.

Patient and public involvement. For the larger parent study in which participants were involved, national and community advisory boards provided input on intervention priorities. Health facility providers, managers, and local authorities were involved in implementation activities and influenced the focus and content of those activities based on their roles and priorities.

While caregiver participants were not involved in the design or conduct of this cross-sectional study, other than being a participant, findings specific to their child's data were disseminated directly to caregivers at the visit. If health conditions or neurodevelopmental delays were identified, clinical referrals were made as well.

Ethical considerations. This project was approved by the Scientific and Ethics Review Unit of the Kenya Medical Research Institute (KEMRI/SERU/CCR/0104/3668) and the University of California San Francisco Institutional Review Board (UCSF IRB#: 18-25555). Written authorization was obtained from the Migori County Director of Health. Formal written informed consent procedures were completed in the preferred language of each caregiver.

Statistical Analysis. Analyses involved the use of descriptive statistics, as well as univariate regression models. Descriptive statistics involved the use of frequencies and proportions for categorical variables, and mean, median, range, inter-quartile range and standard deviation for continuous variables. Socio-demographic and clinical factors associated with neurodevelopmental delay and malnutrition in infants were examined in univariate logistic regression models using the total dataset without age categorization due to small sample size.

For peer review only

Risk of neurodevelopmental delay or malnutrition was computed as an odds ratio with a confidence level of 95%. All analyses were completed using STATA Version 13.0 Stata/MP.

Child medical experiences were summarized as past medical illnesses (since birth) or current medical status (within 2 weeks of the assessment). MDAT and HINE total and domain scores were calculated. MDAT scores were investigated using 2 methods. First, a child was noted to have failed the MDAT overall if they were unable to complete 2 or more items in any one domain that would be expected to be passed by 90% of the normal reference population at their age.(18) Second, developmental z-scores were calculated using the most current MDAT Scoring Application (beta test version v1.1), and scores were dichotomized as either typical (> -2 standard deviations (SD) of mean) or delayed (≤ -2 SD to mean). For the HINE, a score of < 64 was used, as this has been shown to be 98% predictive of walking at 2 years with a sensitivity of 85% for PT children.(19) TQQ findings were described per age group, with overall caregiver concern noted if one or more items were endorsed. Apart from each assessment's categorization of neurodevelopmental delay, a composite dichotomous neurodevelopmental delay variable was created, with a child considered to have delay if their score met delay criteria on at least one of the three neurodevelopmental tools.

For growth, World Health Organization (WHO) child growth standards were used in calculation of z-scores as provided in the STATA igrowup package.(20) Nutritional status z-scores of weight for age (WAZ), length for age (LAZ) and weight for length (WLZ) were calculated.(21) Outcomes were categorized into normal (≥-1 for WAZ and LAZ; ≥-1 to ≤ 2 for WLZ), at risk (≥-2 to <-1), moderate (< -2 to ≥ -3) or severe (< -3). Overweight and obese were defined as WLZ >2 to ≤ 3 and WLZ > 3, respectively. A composite dichotomous malnutrition

For peer review only

variable was created with those meeting moderate or severe criteria in at least one of the three nutritional z-score variables considered malnourished.

All available data were included in the analyses. There were few missing datapoints, and any cases of missingness for pregnancy, infant and child health characteristics are noted in Tables 1 and 2. No datapoints were missing for the MDAT or the HINE. One 12-month-old did not have a complete TQQ. Records with missing data were omitted only for each respective analysis.

RESULTS

Of 761 eligible infants, 564 (74.1%) of caregivers were located. A total of 28 infants (3.7% of eligible) had died after 28 days of life and prior to study contact. While the specific causes of death for these infants are not known, a larger verbal and social autopsy study of the full parent study sample was conducted.(22) Of the 382 live babies consented for assessment (50.2% of eligible infants), six were not assessed due to acute illness at the time of appointment. The final sample included in analysis consisted of 362 infants (47.6% of eligible infants) with viable data, of which 155, 159 and 48 were 6, 12 and 18 months of age respectively (Figure 1). The target sample size of 183 per age group was not reached due to the parent study ending earlier than expected and a national health worker strike that particularly restricted the pool of eligible 18-month-olds.

Characteristics at Delivery and Immediate Postnatal Period

Most babies were female (60.2%) and moderate to late PT (56.6%, >32 weeks' gestation; median gestational age and range = 36.3 weeks (22.0, 41.7). Of infants born preterm, 66.1% were late preterm (34 to <37 weeks), 17.6% were moderate preterm (32 to <34 weeks), 13.9% were very preterm (28 to <32), and only 2.5% were extremely preterm (22 to <28). Birthweight

For peer review only

was over 2500g for 35.6%, and more than 90% had 5-minute Apgar scores ≥7. Sixteen percent were admitted to the newborn unit, and 35.6% needed special care (i.e., oxygen, phototherapy, kangaroo mother care) in the first month of life. Approximately 50.6% of mothers were aged 19 to 25. Most were multiparous (70.4%), and 13% of deliveries were by C-section (Table 1).

Table 1: Delivery and Immediate Postnatal Period Characteristics

Age at Assessment	6 months n (%)	12 months n (%)	18 months n (%)	All n (%)
Neonatal factors				
Gender				
Male	57 (36.8)	64 (40.3)	23 (47.9)	144 (39.8)
Female	98 (63.2)	95 (59.8)	25 (52.1)	218 (60.2)
Multiple pregnancy (Twins)	50 (32.3)	46 (28.9)	10 (20.8)	106 (29.3)
Gestational Age (weeks)				
≥ 37*	59 (38.1)	45 (28.3)	10 (20.8)	114 (31.5)
34 to <37	60 (38.7)	78 (49.1)	24 (50.0)	162 (44.8)
32 to <34	16 (10.3)	18 (11.3)	9 (18.8)	43 (11.9)
28 to <32	17 (11.0)	12 (7.6)	5 (10.4)	34 (9.4)
22 to <28	3 (1.9)	3 (1.9)	0	6 (1.7)
Unknown	0	3 (1.9)	0	3 (0.8)
Birthweight (grams)				
2500 – 2999**	50 (32.3)	58 (36.5)	21 (43.8)	129 (35.6)
1500 – 2499	94 (60.7)	97 (61.0)	27 (56.2)	218 (60.2)
1000 – 1499	7 (4.5)	3 (1.9)	0	10 (2.8)
500 – 999	4 (2.6)	1 (0.6)	0	5 (1.4)
Apgar – 5-minute				
0 to 3	0	0	1 (2.1)	1 (0.3)
4 to 6	7 (4.5)	4 (2.5)	0	11 (3.0)
>= 7	141 (91.0)	144 (90.6)	47 (97.9)	332 (91.7)
Unknown	7 (4.5)	11 (6.9)	0	18 (5.0)
Admitted to Newborn Unit (Yes)	28 (18.1)	22 (13.8)	8 (16.7)	58 (16.0)
"Special care" in first month (Yes)	59 (38.0)	59 (37.1)	11 (22.9)	129 (35.6)
Oxygen	19 (32.2)	8 (13.6)	3 (27.3)	30 (23.3)
Phototherapy	3 (5.1)	4 (6.8)	0 (0)	7 (5.4)
Kangaroo Mother Care	52 (88.1)	56 (89.8)	10 (90.9)	118 (91.5)
Maternal factors				
Age (years)				
< 19	24 (15.5)	12 (7.6)	5 (10.4)	41 (11.3)
19 to 25	71 (45.8)	85 (53.5)	27 (56.3)	183 (50.6)
> 25	60 (38.7)	62 (39.0)	16 (33.3)	138 (38.1)
Parity				

For peer review only

Primigravida	51 (32.9)	46 (28.9)	10 (20.8)	107 (29.6)
Multigravida	104 (67.1)	113 (71.1)	38 (79.2)	255 (70.4)
Delivery Mode				
Vaginal	125 (80.7)	144 (90.6)	40 (83.3)	309 (85.4)
Cesarean	26 (16.8)	14 (8.8)	8 (16.7)	48 (13.3)
Unknown	4 (2.6)	1 (0.1)		5 (13.8)

* Infants ≥ 37 weeks' gestation were included only if birthweight was < 2500 grams.
** Infants 2500 – 2999 grams were included only if gestational age was < 37 weeks.

Compared to the eligible pool of caregivers and infants from the parent study, mothers in the current study were older on average (24.7 years vs. 23.6 years, t=3.16, p<0.005), and babies were more likely female (60.2% vs. 52.8%, χ^2=7.73, p=0.02). The two groups did not differ significantly in other key demographic variables (Supplemental Table 3).

Growth and Health

Anthropometric measurement and caregiver-reported health findings are in Tables 2 and 3. The prevalence of stunting, underweight, and wasting in the study population were 27.4%, 17.2% and 3.3%, respectively. The proportions of children with malnutrition increased with infant age. Moderate to severe malnutrition was significantly more common in males than females (OR 2.53, 95% CI 1.62-3.97), and in babies born after multiple gestation (OR 1.72, 95% CI 1.08-2.75) or with birthweight 1500 to 2499g (OR 1.73, 95% CI 1.07-2.81).

The most common illnesses reported as ever experienced by participants included malaria (56.7%), diarrheal disease (55.2%) serious febrile illness (42.3%); and in the past 2 weeks prior to assessment, respiratory tract infections (26%).

For peer review only

Table 2. Child Characteristics at Time of Visit

Age at Assessment	6 months<br>n (%)	12 months<br>n (%)	18 months<br>n (%)	All<br>n (%)
Weight for Age Z-score (WAZ; Underweight; valid n=343) *				
Normal	87 (58.4)	68 (45.3)	27 (61.4)	182 (53.1)
At risk	43 (28.9)	52 (34.7)	7 (15.9)	102 (29.7)
Moderate	13 (8.7)	20 (13.3)	7 (15.9)	40 (11.7)
Severe	6 (4.0)	10 (6.7)	3 (6.8)	19 (5.5)
Length for Age Z-score (LAZ; Stunting; valid n=351) *				
Normal	71 (46.7)	62 (40.5)	20 (43.5)	153 (43.6)
At risk	47 (30.9)	47 (30.7)	8 (17.4)	102 (29.1)
Moderate	24 (15.8)	26 (17.0)	11 (23.9)	61 (17.4)
Severe	10 (6.6)	18 (11.8)	7 (15.2)	35 (10.0)
Weight for Length Z-score (WLZ; Wasting; valid n=339)*				
Normal	107 (73.3)	89 (59.7)	31 (70.5)	227 (67.0)
At risk	22 (15.1)	38 (25.5)	7 (15.9)	67 (19.8)
Moderate	5 (3.4)	11 (7.4)	4 (9.1)	20 (5.9)
Severe	5 (3.4)	7 (4.7)	2 (4.6)	14 (4.1)
Overweight	6 (4.1)	4 (2.7)	0	10 (3.0)
Obese	1 (0.7)	0	0	1 (0.3)
Composite Malnutrition (Underweight/Stunted/Wasting)**				
Normal	111 (71.6)	100 (62.9)	26 (54.2)	237 (65.5)
Malnourished	44 (28.4)	59 (37.1)	20 (41.7)	123 (34.0)
Missing	0	0	2 (4.2)	2 (0.6)
Past Medical Illnesses (birth until study evaluation)				
Pneumonia	9 (5.8)	13 (8.2)	6 (12.5)	28 (7.7)
Diarrheal Disease	63 (40.7)	107 (67.3)	30 (62.5)	200 (55.2)
Seizures	8 (5.2)	22 (13.8)	4 (8.3)	34 (9.4)
Malaria	55 (35.5)	107 (67.3)	43 (89.6)	205 (56.7)
Serious febrile illness/ meningitis	28 (58.3)	41 (26.5)	84 (52.8)	153 (42.3)
Cough for > 2 weeks	13 (8.4)	26 (16.4)	5 (10.4)	44 (12.2)
Malnutrition	2 (1.3)	3 (1.9)	3 (6.2)	8 (2.2)
Skin infections	26 (16.8)	51 (32.1)	15 (31.3)	92 (25.4)
Current Medical Illness (in past 2 weeks)				
Acute febrile illness	3 (1.9)	1 (0.6)	0	4 (1.1)
Gastroenteritis/dysentery	21 (13.5)	20 (12.6)	6 (12.5)	47 (13.0)
Acute Malnutrition	2 (1.3)	2 (1.3)	0	4 (1.1)
Respiratory tract infection/ pneumonia	33 (21.3)	48 (30.2)	13 (27.1)	94 (26.0)
Others ***	17 (11.4)	18 (11.3)	5 (10.4)	40 (11.0)
Referred for further care	13 (8.4)	15 (11.3)	5 (10.4)	33 (9.1)

* Normal (≥-1 for WAZ and LAZ; ≥-1 to ≤ 2 for WLZ), At risk (≥-2 to <-1), Moderate (< -2 to ≥ -3), Severe (< -3). Overweight WLZ >2 to ≤ 3, Obese WLZ > 3

For peer review only

** Composite malnutrition includes infants who were either underweight, stunted or wasted.
*** Other illnesses included abscess (1), thrush (4), scabies (8), dermatitis (3), skin infection (18), anemia (1), convulsions (3), otitis media (1), congenital cataract (1)

Table 3. Univariate analyses for malnutrition

Infant and maternal variables	Malnutrition (underweight)	Malnutrition (stunting)	Malnutrition (wasting)	Malnutrition (under/stunt/wast)
Gender				
Male	2.52 (1.42,4.48)**	2.98 (1.83,4.83)***	1.54 (0.76,3.14)	2.53 (1.62,3.97)***
Female	1.0	1.0	1.0	1.0
Mothers Age				
<19	0.83 (0.32,2.15)	0.71 (0.32,1.60)	1.84 (0.67,5.10)	1.00 (0.49,2.03)
19-25	1.0	1.0	1.0	1.0
>25	0.99 (0.55,1.80)	0.89 (0.54,1.47)	1.10 (0.50,2.40)	0.99 (0.62,1.58)
Multiple pregnancy				
Yes	1.98 (1.11,3.53)*	1.45 (0.88,2.39)	1.48 (0.71,3.08)	1.72 (1.08,2.75)*
No	1.0	1.0	1.0	1.0
Mode of delivery				
VD	1.0	1.0	1.0	1.0
CS	1.02 (0.45,2.32)	0.71 (0.34,1.51)	1.76 (0.72,4.31)	0.95 (0.50,1.81)
Apgar1 score				
≤5	0.29 (0.09, 0.93)*	0.45 (0.15,1.34)	0.59 (0.12,2.79)	0.35 (0.12,1.05)
6 - 7	0.76 (0.19,2.98)	1.24 (0.34,4.50)	0.92 (0.15,5.78)	1.06 (0.29,3.86)
>7	1.0	1.0	1.0	1.0
Apgar2 score				
≤5	0.20 (0.03,1.46)	0.56 (0.09,3.44)	0.33 (0.03,3.26)	0.35 (0.06,2.14)
6 - 7	0.44 (0.05,4.37)	0.94 (0.11,7.73)	0.55 (0.04,8.27)	0.57 (0.07,4.64)
>7	1.0	1.0	1.0	1.0
GA				
≥37	1.0	1.0	1.0	1.0
33 to <37	0.90 (0.49,1.66)	0.98 (0.59,1.64)	0.61 (0.28,1.30)	1.00 (0.16,1.61)
28 to < 33	0.61 (0.19,1.94)	0.77 (0.32,1.88)	0.43 (0.09,1.99)	0.77 (0.34,1.77)
22 to <28	2.97 (0.46,18.94)	PF	4.43 (0.68,28.89)	0.93 (0.62,5.27)
Birth Weight				
2500 – 2999	1.0	1.0	1.0	1.0
1500 – 2499	1.38 (0.74,2.55)	1.86 (1.10,3.18)*	1.31 (0.60,2.86)	1.73 (1.07,2.81)*
1000 – 1499	0.65 (0.08,5.49)	3.44 (0.97,12.21)	PF	2.35 (0.67,8.21)
500 - 999	1.47 (0.15,13.96)	1.03 (0.11,9.65)	2.65 (0.27,26.04)	1.88 (0.30,11.74)
HINE				
Normal	1.0	1.0	1.0	1.0
Delayed	3.75 (0.82,17.22)	1.06 (0.22,5.58)	7.28 (1.56,34.03)*	1.46 (0.32,6.61)
MDAT (Pass/Fail)				
Normal	1.0	1.0	1.0	1.0
Delayed	4.08 (1.63, 10.19)**	3.50 (1.46, 8.40)**	2.38 (0.75, 7.59)	3.21 (1.35, 7.65)**
MDAT (z-scores)				
Normal	1.0	1.0	1.0	1.0
Delayed	6.48 (1.69, 24.92)**	4.18 (1.15, 15.16)*	4.77 (1.14, 20.04)*	8.07 (1.69, 38.61)**
TQQ				
Normal	1.0	1.0	1.0	1.0
Delay	2.03 (1.09, 3.78)*	0.97 (0.55, 1.71)	2.24 (1.05, 4.80)*	1.40 (0.84, 2.34)

*** p-value < 0.001
** p-value < 0.01
* p-value <0.05

For peer review only

PF – No variability due to low numbers causes the model to perfectly predict failure or success.

Neurodevelopment

Delays on one or more of the standardized neurodevelopmental assessment tools were identified in 8.6% of infants (Tables 4 and 5). The 12-month-old infants were more likely to show delays than infants of the other two age groups, with gross motor and personal-social (MDAT z-score) areas most impacted. Seven children (1.9%) showed HINE findings indicative of cerebral palsy. In univariate analysis, a HINE score concerning for cerebral palsy was more likely in children born by C-section (OR 9.27, 95% CI 2.0-42.8) and was significantly associated with wasting (OR 7.28, 95% CI 1.56-34.03). Neurodevelopmental delay was more likely in males (OR 3.55, 95% CI 1.62-7.79) and in infants who were underweight (OR 4.01, 95% CI 1.80-8.94), stunted (OR 2.96, 95% CI 1.39-6.33), or wasted (OR 2.76, 95% CI 1.03-7.36). Overall, 22.7% of caregivers expressed some concern about their child's neurodevelopment on the TQQ.

Table 4. Neurodevelopmental Outcomes

Age at Assessment	6 months n (%)	12 months n (%)	18 months n (%)	All n (%)
Delayed by MDAT†				
Pass/Fail criteria				
Gross Motor	6 (3.9)	9 (5.7)	0	15 (4.1)
Fine Motor	1 (0.7)	2 (1.3)	1 (2.1)	4 (1.1)
Language	0	2 (1.3)	1 (2.1)	3 (0.8)
Personal Social	1 (0.7)	2 (1.3)	1 (2.1)	4 (1.1)
Total MDAT*	8 (5.2)	12 (7.6)	3 (6.3)	23 (6.4)
<= -2 SD from Mean				
Gross Motor	0	10 (6.3)	0	10 (2.8)
Fine Motor	6 (3.9)	4 (2.5)	1 (2.1)	11 (3.0)
Language	5 (3.2)	3 (1.9)	2 (4.2)	10 (2.8)
Personal Social	3 (1.9)	15 (9.4)	0	18 (5.0)
Total MDAT*	2 (1.3)	6 (3.8)	2 (4.2)	10 (2.8)

For peer review only

Delayed by HINE†	5 (3.2)	1 (0.6)	1 (2.1)	7 (1.9)
Neurodevelopmental Delay††	12 (7.7)	15 (9.4)	4 (8.3)	31 (8.6)
Ten Questions Questionnaire: Total with one or more concerns	18 (11.6)	43 (27.0)	21 (43.8)	82 (22.7)

† MDAT=Malawi Developmental Assessment Tool; HINE = Hammersmith Infant Neurologic Examination
†† Neurodevelopmental Delay defined as a fail on one or more of the 3 evaluation criteria, MDAT Pass/Fail, MDAT Z-score (≤ -2 standard deviations from mean) or HINE.
* NOTE: A fail score on the total MDAT can occur with a fail in any one or more subscales, thus this number does not represent the sum of children failing on the domain scores.

Table 5. Univariate analyses for neurodevelopmental delay

Infant and maternal variables	Neurodevelopmental delay (HINE)	Neurodevelopmental delay (TQQ)	Neurodevelopmental delay (MDAT Pass/Fail)	Neurodevelopmental delay (MDAT z-scores)	Neurodevelopmental delay (HINE/MDAT pass/fail or z-scores)
	OR (95% CI)	OR (95% CI)	OR (95% CI)	OR (95% CI)	OR (95% CI)
Gender					
Male	3.88 (0.74,20.30)	0.95 (0.58, 1.58)	3.71 (1.49, 9.27)**	3.61 (0.92, 14.20)	3.55 (1.62,7.79)**
Female	1.0	1.0	1.0	1.0	1.0
Mothers Age					
<19	3.08 (0.50,19.04)	0.56 (0.23, 1.35)	0.73 (0.16, 3.38)	2.79 (0.64, 12.20)	1.45 (0.50,4.21)
19-25	1.0	1.0	1.0	1.0	1.0
>25	0.88 (0.15,5.35)	0.64 (0.37, 1.10)	1.00 (0.41, 2.46)	0.53 (0.10, 2.77)	0.82 (0.36,1.86)
Multiple pregnancy					
Yes	3.26 (0.72,14.83)	1.15 (0.68, 1.97)	1.06 (0.42, 2.66)	1.04 (0.26, 4.09)	1.35 (0.62,2.92)
No	1.0	1.0	1.0	1.0	1.0
Mode of delivery					
VD	1.0	1.0	1.0	1.0	1.0
CS	9.27 (2.01,42.82)**	1.36 (0.68, 2.71)	1.00 (0.29, 3.52)	3.00 (0.75, 12.04)	2.03 (0.82,5.00)
Apgar score at 1 min					
≤5	0.12 (0.02,0.55)**	1.07 (0.29, 3.96)	0.31 (0.06, 1.53)	0.26 (0.03, 2.36)	0.45 (0.09,2.14)
>6	1.0	1.0	1.0	1.0	1.0
Apgar score at 5 min					
≤5	PF	1.20 (0.13, 10.90)	0.38 (0.08, 1.82)	0.34 (0.04, 2.91)	0.54 (0.11,2.54)
>6	1.0	1.0	1.0	1.0	1.0
GA					
≥37	1.0	1.0	1.0	1.0	1.0

For peer review only

33 to <37	0.74 (0.16,3.35)	1.10 (0.63, 1.90)	1.12 (0.44, 2.86)	1.12 (0.27, 4.55)	1.00 (0.45,2.25)
28 to < 33	PF	1.10 (0.44, 2.72)	0.96 (0.19, 4.83)	1.12 (0.11, 11.14)	1.01 (0.26,3.89)
22 to <28	PF	PF	PF	PF	PF
Birth Weight					
2500 – 2999	1.0	1.0	1.0	1.0	1.0
1500 – 2499	3.59 (0.43,30.20)	1.11 (0.66, 1.87)	1.50 (0.57, 3.96)	0.89 (0.25, 3.20)	2.45 (0.97,6.19)
1000 – 1499	PF	1.55 (0.38, 6.37)	5.04 (0.87, 29.10)	PF	5.17 (0.90,29.81)
500 - 999	PF	PF	PF	PF	PF
Underweight					
Normal	1.0	1.0	1.0	1.0	1.0
Abnormal	3.75 (0.82,17.22)	2.03 (1.09, 3.78)*	4.08 (1.63, 10.19)**	6.48 (1.69, 24.92)**	4.01 (1.80,8.94)**
Stunting					
Normal	1.0	1.0	1.0	1.0	1.0
Abnormal	1.06 (0.20,5.58)	0.97 (0.55, 1.71)	3.50 (1.46, 8.40)**	4.18 (1.15, 15.16)*	2.96 (1.39,6.33)**
Wasting					
Normal	1.0	1.0	1.0	1.0	1.0
Abnormal	7.28 (1.56,34.03)*	2.24 (1.05, 4.80)*	2.38 (0.75, 7.59)	4.77 (1.14, 20.04)*	2.76 (1.03, 7.36)*

*** p-value < 0.001
** p-value < 0.01
* p-value <0.05
PF – No variability due to low numbers causes the model to perfectly predict failure or success.

As described previously, this study recruited infants who had participated in a cRCT. The number of infants with neurodevelopmental delay were small in both control and intervention groups, and the sample was not large enough to be adequately powered to detect significant group differences if present. These data are provided for review in Supplemental Tables 4 and 5, but should be interpreted with caution.

DISCUSSION

This study describes growth and neurodevelopmental outcomes for a rural community sample of PT/LBW survivors. Infants were similar in gestational age to other community-based samples from countries with NMR ≥ 5 and constituted a relatively low-risk sample of PT/LBW infants compared to high-resource contexts or LMIC settings with available NICU care. Only 27% were born at the county's tertiary referral hospital, with the remaining born at other rural

For peer review only

facilities. Surviving infants would thus be expected to have better outcomes than their counterparts requiring neonatal intensive care in urban settings of Africa.

Rates of stunting and underweight were higher than locally reported data, suggesting a higher risk of malnutrition in the current PT/LBW sample than in general population of young children in the local community. Direct comparison to growth data from available community-based African samples is complicated by differences in country under-5 malnutrition rates when these studies took place.(13,23,24) Nonetheless, findings are concerning, particularly given low parental awareness (fewer than 3% expressed concern for acute or chronic malnutrition) and apparently limited detection/intervention at routine child health/immunization visits. These findings suggest that future work focused on caregiver understanding of appropriate growth in infants born PT or LBW will be important to assuring early detection and management.

This study demonstrates that standardized assessments can be locally implemented to enhance neurodevelopmental evaluation at the community level. Directly administered, standardized neurodevelopmental assessment tools identified delay or disability in 8.6% of PT/LBW infants. This proportion is lower than global estimates from settings with high NMR but NICU care available, where one might anticipate higher risk infants surviving. It is more comparable to, but still lower than that of other cited community-based studies.(3,8,10) A higher number of caregivers expressed developmental concerns, with more concern for older children, likely in part due to the increase in observable developmental milestones/skills as children age.

The HINE was successfully used as a predictive assessment for cerebral palsy or motor disability. Approximately 2% of children showed concern for being non-ambulatory by 2 years, and one additional child met clinical criteria for cerebral palsy but was not included in the sample due acute illness at the time of visit. While these numbers are low, the percentage is not

For peer review only

markedly different than the 3.4% of children with neonatal encephalopathy who had "sub-optimal" HINE scores in a recent Ugandan study.(25) With global PT births estimated at 15 million annually, even these small percentages would translate to almost 1.3 million children with developmental delay or high risk for disability annually, highlighting the importance of targeted clinical follow-up and implementation of early intervention programs for these at-risk infants in low-resource communities.(12,26)

In addition to malnutrition and neurodevelopmental risks, a high proportion of the sample were reported to have experienced acute childhood illness in their lifetime, including malaria, diarrheal disease, and serious febrile illness. Children in the current study had higher rates of acute respiratory infection in the last two weeks than local averages for children under 5 years (26% vs. 13%).(13) Increased rates of respiratory and severe infections have been documented for PT infants in other contexts, indicating that these major illnesses may differentially affect PT/LBW infants.(10) Although community data for the other illnesses are lacking, malaria is endemic in Migori County and a major cause of under-5 mortality (19%).(27)

Our data may underestimate true developmental delay/disability rates for PT/LBW infants for two reasons. First, participants were part of a larger cRCT evaluating the effect of an intrapartum and immediate postnatal intervention package on PT/LBW neonatal survival in which the control arm also received two of the four interventions. Post-hoc univariate analyses revealed no significant differences in growth or neurodevelopment between babies born at control versus intervention sites (Supplemental Tables 4 and 5); however, these findings should be considered with caution due to the small sample size and because this cross-sectional study was not designed to evaluate the impact of the cRCT intervention on growth or neurodevelopmental outcomes.(11,12) Second, study participants were largely moderate to late

For peer review only

PT infants with predominately normal or LBW, as opposed to very or extremely PT/LBW infants, and the vast majority had 5-minute Apgar scores ≥ 7.(28) These findings are consistent with WHO data suggesting that half of babies born before 32 weeks in low-income countries will not survive; however, they suggest findings may be an underestimate of adverse outcomes of PT/LBW babies in LMIC more broadly.(29) Compared to all infants who survived to 28 days in the larger parent study, infants in this sample were more likely to be female and to have younger mothers at time of delivery (Supplemental Table 3). Since 79% of infants who died prior to study contact were female, survival bias is an unlikely reason for this female predominance. However, in our small sample, males were more likely to be malnourished and have developmental delay, suggesting that additional longitudinal investigation into gender-related outcomes is warranted. Whether maternal age differences were due to differential survival or challenges in locating teen mothers is unknown; however, future research would ideally gather information on surviving PT infants among adolescent mothers in LMIC. Other important sample characteristics did not differ, suggesting the sample was largely representative of the PT/LBW population. In contrast, there is the possibility that these data may bias somewhat toward higher risk of health, growth, and neurodevelopmental difficulties, since almost 30% of participating infants were born after twin pregnancies. Future studies with long-term follow-up of PT/LBW infants may consider including only singleton births or planning a priori for additional analyses comparing infants from singleton pregnancies with those born after twin pregnancies.

This study has several limitations. First, the study design did not allow for direct comparison to term, normal birthweight controls, and it was not possible to investigate factors contributing to poor growth or neurodevelopmental outcomes through multivariate analyses. Additionally, there were too few babies in the highest-risk PT/LBW categories to separately

For peer review only

investigate their neurodevelopmental outcomes. There were several constraints related to recruitment for this study. The parent study was not originally designed as a longitudinal follow up past 28 days, and this meant that we did not have recurrent contact with caregivers between the infant turning 28 days and the follow-up study recruitment call, which occurred up to 17 months later. Additionally, a national health worker strike significantly reduced recruitment into the parent study during the birth months of 18-month-olds, markedly reducing the number of potentially eligible children at this age. Although only 6.9% of those contacted declined to participate, 25.9% were unreachable, and 20.4% of those scheduled for an informational recruitment visit did not attend that visit, so it was not possible to describe the study to them in detail. The analyzed sample consisted of just under 50% of the identified eligible sample (Figure 1), suggesting possible selection bias in this subsample. Despite these limitations in sampling, our data contribute to the very limited follow-up data on outcomes in PT/LBW infants in community samples of LMIC. The experienced challenges in recruitment underscore the importance of setting up robust longitudinal cohorts to obtain high quality data on the long-term outcomes of these vulnerable infants in LMIC to inform intervention and policy planning.

Conclusion

The current study adds to very limited community-based literature on PT/LBW infants born in countries with high NMR and suggests higher than background rates of wasting and underweight, high rates of parental concern for development, and a clinically impactful number of children with neurodevelopmental delay or risk for disability. The results highlight the need for policies that support close monitoring of and early intervention for high-risk infants to assure PT/LBW infants in both rural and urban areas of LMIC are able to thrive.

For peer review only

Acknowledgements: We wish to thank the caregivers and infants who generously gave their time to participate in this project.

Funding Source: This work was supported by the Bill & Melinda Gates Foundation under grant number OPP1107312 (East Africa Preterm Birth Initiative).

Ethics Approval: This project was approved by the Scientific and Ethics Review Unit (SERU) of the Kenya Medical Research Institute (SERU #: KEMRI/SERU/CCR/0104/3668) and the UCSF Institutional Review Board (UCSF IRB#: 18-25555). Written authorization was obtained from the Migori County Director of Health.

Data Availability: The data that support the findings of this study are available from the corresponding author, Susanne Martin-Herz, upon reasonable request.

Word Count: 3106 (4239 including Tables)

Competing interests: The authors have no financial relationships or conflicts of interest relevant to this article to disclose.

Contributorship statement: SMH, PO, GN, NS and DW contributed to study conceptualization. VM, GO led data cleaning and analysis. SMH, PO, GN, VM, GO, NS and DW contributed to data synthesis and interpretation. SMH and PO drafted and revised the manuscript, GN, VM, GO, NS and DW provided edits and feedback. All authors approved the final version of the manuscript for submission.

For peer review only

REFERENCES

1. World Health Organization. WHO Newborn Deaths and Illnesses. Published online 2011. Accessed March 20, 2018. http://www.who.int.ucsf.idm.oclc.org/pmnch/media/press_materials/fs/fs_newborndealth_illness/en/

2. Ahmed I, Ali SM, Amenga-Etego S, et al. Population-based rates, timing, and causes of maternal deaths, stillbirths, and neonatal deaths in south Asia and sub-Saharan Africa: a multi-country prospective cohort study. *The Lancet Global Health*. 2018;6(12):e1297-e1308. doi:10.1016/S2214-109X(18)30385-1

3. Blencowe H, Lee ACC, Cousens S, et al. Preterm birth-associated neurodevelopmental impairment estimates at regional and global levels for 2010. *Pediatric research*. 2013;74 Suppl 1(Journal Article):17.

4. Lawn JE, Blencowe H, Oza S, et al. Every Newborn: Progress, priorities, and potential beyond survival. *The Lancet*. 2014;384(9938):189-205.

5. Murray CJL Prof, Vos T Prof, Lozano R Prof, et al. Disability-adjusted life years (DALYs) for 291 diseases and injuries in 21 regions, 1990–2010: a systematic analysis for the Global Burden of Disease Study 2010. *Lancet, The*. 2012;380(9859):2197-2223. doi:10.1016/S0140-6736(12)61689-4

6. Gladstone M, Oliver C, Van den Broek N. Survival, morbidity, growth and developmental delay for babies born preterm in low and middle income countries - a systematic review of outcomes measured. *PloS one*. 2015;10(3):e0120566. doi:10.1371/journal.pone.0120566

7. World Health Organization. *World Health Statistics Overview 2019: Monitoring Health for the Sustainable Development Goals*. World Health Organization; 2019. Accessed July 15, 2020. https://apps.who.int/iris/bitstream/handle/10665/311696/WHO-DAD-2019.1-eng.pdf?ua=1

8. Namazzi G, Tumwine JK, Hildenwall H, et al. Neurodevelopmental outcomes of preterm babies during infancy in Eastern Uganda: a prospective cohort study. *Glob Health Action*. 2020;13(1):1820714. doi:10.1080/16549716.2020.1820714

9. Kirk CM, Uwamungu JC, Wilson K, et al. Health, nutrition, and development of children born preterm and low birth weight in rural Rwanda: a cross-sectional study. *BMC Pediatr*. 2017;17(1):191. doi:10.1186/s12887-017-0946-1

10. Gladstone M, White S, Kafulafula G, Neilson JP, van den Broek N. Post-neonatal mortality, morbidity, and developmental outcome after ultrasound-dated preterm birth in rural Malawi: a community-based cohort study. *PLoS medicine*. 2011;8(11):e1001121. doi:10.1371/journal.pmed.1001121

11. Otieno P, Waiswa P, Butrick E, et al. Strengthening intrapartum and immediate newborn care to reduce morbidity and mortality of preterm infants born in health facilities in Migori

For peer review only

County, Kenya and Busoga Region, Uganda: a study protocol for a randomized controlled trial. *Trials*. 2018;19(1):1-12. doi:10.1186/s13063-018-2696-2

12. Walker D, Otieno P, Butrick E, et al. Impact of an intrapartum and immediate newborn care quality improvement package on fresh stillbirth and neonatal mortality among preterm and low birthweight births in Kenya and Uganda: a cluster randomised facility-based trial. *Lancet Global Health*. 2020;8:e1061-70.

13. Kenya National Bureau of Statistics, DHS Program, ICF International. *Kenya 2014 Demographic and Health Survey*.; 2014. Accessed April 5, 2020. https://dhsprogram.com/pubs/pdf/fr308/fr308.pdf

14. Were FN, Bwibo NO. Neonatal nutrition and later outcomes of very low birth weight infants at Kenyatta National Hospital. *African Health Sciences*. 2007;7(2):108-114.

15. Sutton PS, Darmstadt GL. Preterm birth and neurodevelopment: a review of outcomes and recommendations for early identification and cost-effective interventions. *Journal of tropical pediatrics*. 2013;59(4):258-265. doi:10.1093/tropej/fmt012

16. Ballot DE, Potterton J, Chirwa T, Hilburn N, Cooper PA. Developmental outcome of very low birth weight infants in a developing country. *BMC pediatrics*. 2012;12(1):11. doi:10.1186/1471-2431-12-11

17. Cochran WG. *Sampling Techniques*. 3rd ed. John Wiley & Sons; 1977.

18. Gladstone M, Lancaster GA, Umar E, et al. The Malawi Developmental Assessment Tool (MDAT): the creation, validation, and reliability of a tool to assess child development in rural African settings. *PLoS medicine*. 2010;7(5):e1000273. doi:10.1371/journal.pmed.1000273

19. Frisone MF, Mercuri E, Laroche S, et al. Prognostic value of the neurologic optimality score at 9 and 18 months in preterm infants born before 31 weeks' gestation. *The Journal of Pediatrics*. 2002;140(1):57-60. doi:10.1067/mpd.2002.119626

20. World Health Organization, United Nation's Children's Fund. *WHO Child Growth Standards and the Identification of Severe Acute Malnutrition in Infants and Children. A Joint Statement*.; 2009. Accessed February 2, 2020. https://apps.who.int/nutrition/publications/severemalnutrition/9789241598163/en/index.html

21. World Health Organization. Global database on child growth. Published online 2018. Accessed April 22, 2020. https://www.who.int/nutgrowthdb/about/introduction/en/index5.html

22. Olack B, Santos N, Inziani M, et al. Causes of preterm and low birth weight neonatal mortality in a rural community in Kenya: evidence from verbal and social autopsy. *BMC Pregnancy Childbirth*. 2021;21(1):536. doi:10.1186/s12884-021-04012-z

For peer review only

23. National Institute of Statistics of Rwanda, Rwanda Ministry of Finance and Economic Planning, Rwanda Ministry of Health, The DHS Program, ICF International. *Rwanda Demographic and Health Survey 2014-2015*.; 2014.

24. National Statistics Office. *Malawi Demographic and Health Survey 2004*. National Statistics Office and ORC Macro; 2005.

25. Tann CJ, Webb EL, Lassman R, et al. Early childhood outcomes after neonatal encephalopathy in Uganda: A cohort study. *EClinicalMedicine*. 2018;6:26-35. doi:10.1016/j.eclinm.2018.12.001

26. Every Preemie Scale. *Kenya Profile of Preterm and Low Birth Weight Prevention and Care*.; 2019. Accessed August 20, 2020. https://www.everypreemie.org/wp-content/uploads/2019/07/Kenya_7.5.19.pdf

27. Starnes JR, Chamberlain L, Sutermaster S, et al. Under-five mortality in the Rongo Sub-County of Migori County, Kenya: Experience of the Lwala Community Alliance 2007-2017 with evidence from a cross-sectional survey. van Wouwe JP, ed. *PLoS ONE*. 2018;13(9):e0203690. doi:10.1371/journal.pone.0203690

28. American College of Obstetricians and Gynecologists' Task Force on Neonatal Encephalopathy. Neonatal encephalopathy and neurologic outcome, Second Edition. *PEDIATRICS*. 2014;133(5):e1482-e1488. doi:10.1542/peds.2014-0724

29. World Health Organization. *Preterm Birth*. World Health Organization; 2018. Accessed July 18, 2020. https://www.who.int/news-room/fact-sheets/detail/preterm-birth

Figure 1: Study flow diagram

For peer review only

Figure 1: Study Flow Diagram

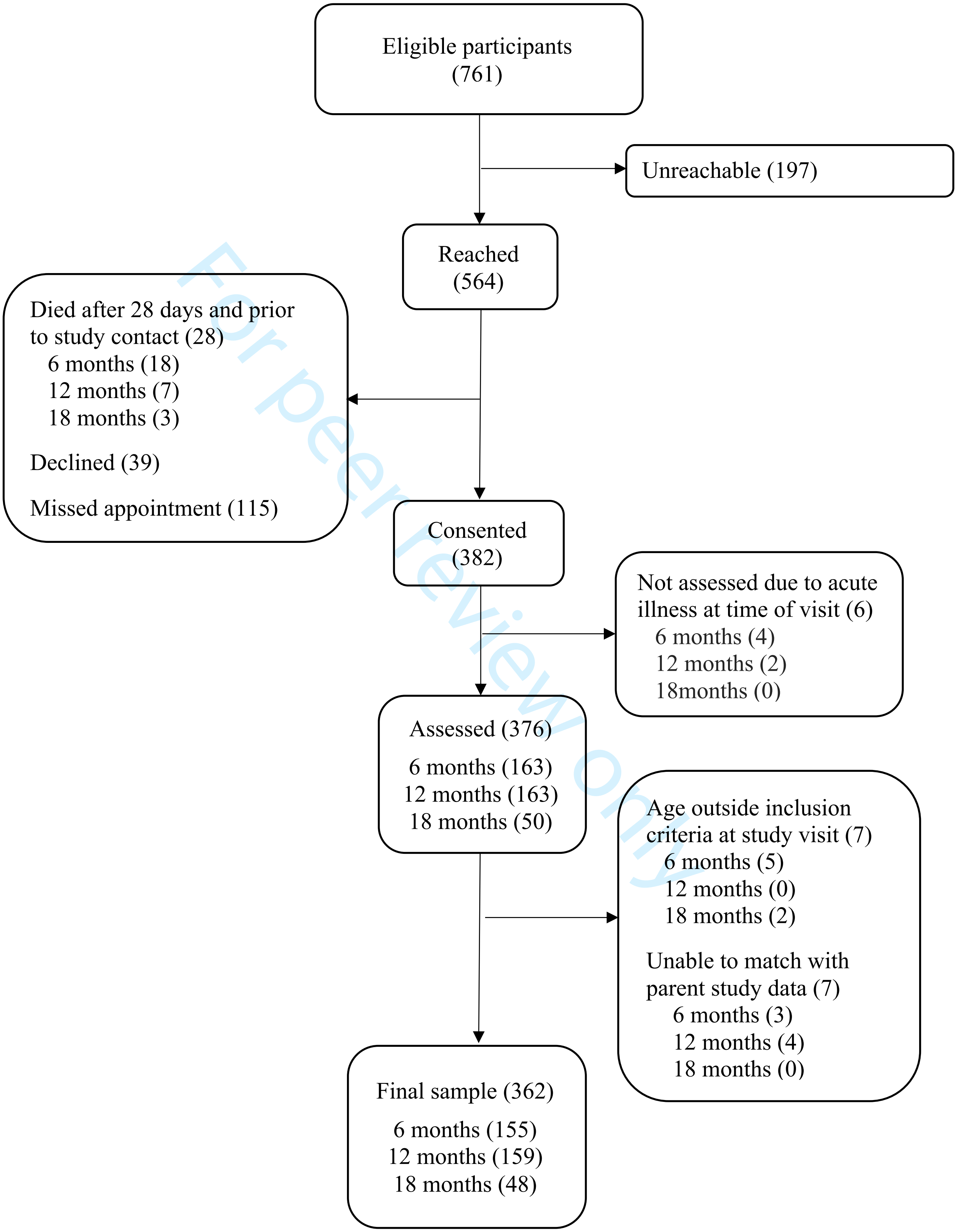

Supplemental Table 1. Anthropometric measurement procedures

Assessment	Methods
Weight	• Digital baby weighing scale calibrated using Standardization weight (10 kg Stone) every morning<br>• Weighing scale surface disinfected before use by the next baby<br>• Weighing scale placed on flat hard surface and made stable before placing baby on<br>• Ensured all clothing removed by caregiver (socks, diapers)<br>• Baby calmed then placed on the weighing scale (sitting or recumbent), unsupported<br>• Measurement read when the scale stopped counting, to the nearest 0.1 kg
Length	• Standard Length Mat placed on a hard flat surface with caregiver as an assistant.<br>• Length Mat surface disinfected before use by the next baby<br>• Ensured all clothing removed by caregiver (socks, diapers)<br>• Caregiver brought the child to the mat and kneeling on the left side and facing the child supported the head and neck to the correct position on mat.<br>• Assessor, kneeling on the right of child, ensured child was in perpendicular position to the base of the length mat, while supporting the knees of child, making sure the shoulders level, hands at child's side, and child's buttocks touching back of length mat<br>• Assessor moved foot piece with right hand until firmly against child's heels<br>• Measurement was read to the nearest 0.1 cm<br>• Procedure was repeated up to 2 times for confirmatory measurement
Mid upper arm circumference (MUAC)	• Used the standard measuring tape that cannot be stretched<br>• With baby on caregiver's lap, assessor exposed and positioned left arm of baby to hang loosely at the side<br>• Shoulder tip identified; tape placed at midpoint and made to run along arm<br>• With elbow flexed, tape positioned on same level, tip of elbow marked and midpoint between tip of the shoulder and tip of bent elbow identified and marked<br>• Adjusting for tension and gaps, tape placed around arm at midpoint and secured using assessor's index finger and thumb at the junction where the 0 mark of the tape meets other end of tape<br>• Measurement recorded to the nearest **0.1cm** and repeated up to 2 times for accuracy, then the average recorded
Occipital Frontal Head Circumference (OFC)	• Used a standard paper measuring tape that cannot be stretched<br>• Securely wrapped tape around widest possible circumference of the head, broadest part of forehead above eyebrow, above ears and most prominent part of back of head<br>• Measurement taken three times<br>• Largest measurement to the nearest **0.1 cm** recorded

For peer review only

Supplemental Table 2. Neurodevelopment Assessment Tools

Assessment	Description	Validity
Ten Questions Questionnaire (TQQ)[31,32]	Brief, caregiver-report screener for neurologic delay or disability. Normed for ages 2 to 9 years and adapted previously for younger children[10]<br>Delay noted if caregiver concern noted on at least one question	Acceptable sensitivity for serious disability[6,33]<br>Successfully used in African contexts[6,33]
Malawi Developmental Assessment Tool (MDAT)[10,34]	Four developmental domains: Gross motor, Fine motor & performance, Language & hearing, Social<br>Developed in Malawi as culturally relevant tool for use in Africa	Excellent reliability and good validity<br>Sensitive to differences between term and preterm infants
Hammersmith Inventory for Neurologic Examination (HINE)[35,36]	Rapid, validated, structured neurologic evaluation	High predictive validity for later cerebral palsy in children from birth to 2 years of age (90% sensitivity)<br>Successfully used in several studies in Africa, as well as clinically in Kenya

For peer review only

Supplemental Table 3. Demographic variables eligible infants from parent study (cRCT) versus enrolled sample for follow-up study

	Parent Study (cRCT) 28-day Survivors n (%)	Follow-up Study Sample n (%)
**Neonatal factors**		
Gender		
Male	1113 (47.0)	144 (39.8)
Female	1255 (53.0)	218 (60.2)
Gestational Age (weeks)		
≥ 37*	989 (36.1)	114 (31.5)
32 to <37	1131 (41.3)	205 (56.6)
28 to <32	183 (6.7)	34 (9.4)
22 to <28	29 (1.1)	6 (1.7)
Unknown	405 (14.8)	3 (0.8)
Birthweight (grams)		
2500 – 2999**	1005 (42.3)	129 (35.6)
1500 – 2499	1282 (54.0)	218 (60.2)
1000 – 1499	74 (3.1)	10 (2.8)
500 – 999	14 (0.6)	5 (1.4)
Apgar – 5 minute		
0 to 3	6 (0.2)	1 (0.3)
4 to 6	84 (3.1)	11 (3.0)
>= 7	2286 (83.5)	332 (91.7)
Unknown	361 (13.2)	18 (5.0)
**Maternal factors**		
Age (years)		
< 19	569 (24.0)	41 (11.3)
19 to 25	1001 (42.3)	183 (50.6)
> 25	797 (33.7)	138 (38.1)
Delivery Mode		
Vaginal	2126 (77.7)	309 (85.4)
Cesarean	230 (8.4)	48 (13.3)
Unknown	381 (13.9)	5 (13.8)

* Infants ≥ 37 weeks' gestation were included only if birthweight was < 2500 grams.
** Infants 2500 – 2999 grams were included only if gestational age was < 37 weeks.
cRCT = cluster randomized control trial

For peer review only

Supplemental Table 4. Child Characteristics **of enrolled sample for follow-up study** by Parent Study (cRCT) Arm

	Intervention n (%)	Control n (%)	All n (%)
Weight for Age Z-score (WAZ; Underweight; valid n=343) *			
Normal	121 (51.9)	61 (55.5)	182 (53.1)
At risk	71 (30.5)	31 (28.2)	102 (29.7)
Moderate	28 (12.0)	12 (10.9)	40 (11.7)
Severe	13 (5.6)	6 (5.5)	19 (5.5)
Length for Age Z-score (LAZ; Stunting; valid n=351) *			
Normal	100 (42.2)	53 (46.5)	153 (43.6)
At risk	81 (34.2)	21 (18.4)	102 (29.1)
Moderate	34 (14.4)	27 (23.7)	61 (17.4)
Severe	22 (9.3)	13 (11.4)	35 (10.0)
Weight for Length Z-score (WLZ; Wasting; valid n=339)*			
Normal	152 (67.3)	75 (66.4)	227 (67.0)
At risk	40 (17.7)	27 (23.9)	67 (19.8)
Moderate	15 (6.6)	5 (4.4)	20 (5.9)
Severe	11 (4.9)	3 (2.7)	14 (4.1)
Overweight	7 (3.1)	3 (2.7)	10 (3.0)
Obese	1 (0.4)	0	1 (0.3)
Composite Malnutrition (Underweight/Stunted/Wasting)**			
Normal	163 (66.5)	74 (63.3)	237 (65.5)
Malnourished	80 (32.7)	43 (36.8)	123 (34.0)
Missing	2 (0.8)	0	2 (0.6)
Past Medical Illnesses (birth until study evaluation)			
Pneumonia	18 (7.4)	10 (8.6)	28 (7.7)
Diarrheal Disease	121 (49.4)	71 (60.7)	192 (53.0)
Seizures	24 (9.8)	10 (8.6)	34 (9.4)
Malaria	141 (57.6)	64 (54.7)	205 (56.6)
Serious febrile illness/meningitis	97 (39.6)	55 (47.0)	152 (42.0)
Cough for > 2 weeks	33 (13.5)	11 (9.4)	44 (12.2)
Malnutrition	5 (2.0)	3 (2.6)	8 (2.2)
Skin infections	60 (24.5)	32 (27.4)	92 (25.4)
Current Medical Illness (in past 2 weeks)			
Acute febrile illness	3 (1.2)	1 (0.9)	4 (1.1)
Gastroenteritis/dysentery	33 (13.5)	14 (12.0)	47 (13.0)
Acute Malnutrition	4 (1.6)	0	4 (1.1)
Respiratory tract infection/pneumonia	76 (31.0)	18 (15.4)	94 (26.0)
Others ***	30 (12.2)	12 (10.3)	42 (11.6)
Referred for further care	27 (11.0)	8 (6.8)	35 (9.7)

For peer review only

* Normal (≥-1 for WAZ and LAZ; ≥-1 to ≤ 2 for WLZ), At risk (≥-2 to <-1), Moderate (< -2 to ≥ -3), Severe (< -3). Overweight WLZ >2 to ≤ 3, Obese WLZ > 3
** Composite malnutrition includes infants who were either underweight, stunted or wasted.
*** Other illnesses included acute conjunctivitis (1), abscess (1), thrush (4), scabies (8), dermatitis (3), skin infection (18), anemia (1), convulsions (3), otitis media (1), congenital cataract (1), worm infection (1)
cRTC = cluster randomized control trial
Note: The current study was not designed to assess the impact of the intervention on these variables. These data are presented for information only.

For peer review only

Supplemental Table 5. Neurodevelopmental Outcomes **of enrolled sample for follow-up study** by Parent Study (cRCT) Arm

	Intervention n (%)	Control n (%)	All n (%)
Delayed by MDAT†			
Pass/Fail criteria			
Gross Motor	11 (4.5)	4 (3.4)	15 (4.1)
Fine Motor	2 (0.8)	2 (1.7)	4 (1.1)
Language	0	3 (2.6)	3 (0.8)
Personal Social	3 (1.2)	1 (0.9)	4 (1.1)
Total MDAT*	14 (5.7)	9 (7.7)	23 (6.4)
<= -2 SD from Mean			
Gross Motor	8 (3.3)	2 (1.7)	10 (2.8)
Fine Motor	8 (3.3)	3 (2.6)	11 (3.0)
Language	6 (2.5)	4 (3.4)	10 (2.8)
Personal Social	13 (5.3)	5 (4.3)	18 (5.0)
Total MDAT*	7 (2.9)	3 (2.6)	10 (2.8)
Delayed by HINE†	6 (2.5)	1 (0.9)	7 (1.9)
Neurodevelopmental Delay††	20 (8.2)	11 (9.4)	31 (8.6)
Ten Questions Questionnaire: Total with one or more concerns	61 (24.9)	21 (18.0)	82 (22.7)

† MDAT=Malawi Developmental Assessment Tool; HINE = Hammersmith Infant Neurologic Examination
†† Neurodevelopmental Delay defined as a fail on one or more of the 3 evaluation criteria, MDAT Pass/Fail, MDAT Z-score (≤ -2 standard deviations from mean) or HINE.
* NOTE: A fail score on the total MDAT can occur with a fail in any one or more subscales, thus this number does not represent the sum of children failing on the domain scores.
cRTC = cluster randomized control trial
Note: The current study was not designed to assess the impact of the intervention on these variables. These data are presented for information only.

For peer review only

STROBE 2007 (v4) Statement—Checklist of items that should be included in reports of *cross-sectional studies*

Section/Topic	Item #	Recommendation	Reported on page #
**Title and abstract**	1	(*a*) Indicate the study's design with a commonly used term in the title or the abstract	1
		(*b*) Provide in the abstract an informative and balanced summary of what was done and what was found	3
**Introduction**			
Background/rationale	2	Explain the scientific background and rationale for the investigation being reported	5-6
Objectives	3	State specific objectives, including any prespecified hypotheses	6
**Methods**			
Study design	4	Present key elements of study design early in the paper	6-7
Setting	5	Describe the setting, locations, and relevant dates, including periods of recruitment, exposure, follow-up, and data collection	7
Participants	6	(*a*) Give the eligibility criteria, and the sources and methods of selection of participants	7
Variables	7	Clearly define all outcomes, exposures, predictors, potential confounders, and effect modifiers. Give diagnostic criteria, if applicable	7-8
Data sources/ measurement	8*	For each variable of interest, give sources of data and details of methods of assessment (measurement). Describe comparability of assessment methods if there is more than one group	7-9
Bias	9	Describe any efforts to address potential sources of bias	7 -8
Study size	10	Explain how the study size was arrived at	7
Quantitative variables	11	Explain how quantitative variables were handled in the analyses. If applicable, describe which groupings were chosen and why	9-11
Statistical methods	12	(*a*) Describe all statistical methods, including those used to control for confounding	9-11
		(*b*) Describe any methods used to examine subgroups and interactions	9-11
		(*c*) Explain how missing data were addressed	11
		(*d*) If applicable, describe analytical methods taking account of sampling strategy	NA
		(*e*) Describe any sensitivity analyses	NA
**Results**			

For peer review only

Participants	13*	(a) Report numbers of individuals at each stage of study—eg numbers potentially eligible, examined for eligibility, confirmed eligible, included in the study, completing follow-up, and analysed	11
		(b) Give reasons for non-participation at each stage	11, Figure 1
		(c) Consider use of a flow diagram	Figure 1
Descriptive data	14*	(a) Give characteristics of study participants (eg demographic, clinical, social) and information on exposures and potential confounders	11-13
		(b) Indicate number of participants with missing data for each variable of interest	Tables 1, 2, 4
Outcome data	15*	Report numbers of outcome events or summary measures	13-18
Main results	16	(*a*) Give unadjusted estimates and, if applicable, confounder-adjusted estimates and their precision (eg, 95% confidence interval). Make clear which confounders were adjusted for and why they were included	NA
		(*b*) Report category boundaries when continuous variables were categorized	NA
		(*c*) If relevant, consider translating estimates of relative risk into absolute risk for a meaningful time period	NA
Other analyses	17	Report other analyses done—eg analyses of subgroups and interactions, and sensitivity analyses	9-11
**Discussion**			
Key results	18	Summarise key results with reference to study objectives	13-20
Limitations	19	Discuss limitations of the study, taking into account sources of potential bias or imprecision. Discuss both direction and magnitude of any potential bias	21-22
Interpretation	20	Give a cautious overall interpretation of results considering objectives, limitations, multiplicity of analyses, results from similar studies, and other relevant evidence	18-22
Generalisability	21	Discuss the generalisability (external validity) of the study results	20-22
**Other information**			
Funding	22	Give the source of funding and the role of the funders for the present study and, if applicable, for the original study on which the present article is based	23

*Give information separately for cases and controls in case-control studies and, if applicable, for exposed and unexposed groups in cohort and cross-sectional studies.

Note: An Explanation and Elaboration article discusses each checklist item and gives methodological background and published examples of transparent reporting. The STROBE checklist is best used in conjunction with this article (freely available on the Web sites of PLoS Medicine at http://www.plosmedicine.org/, Annals of Internal Medicine at http://www.annals.org/, and Epidemiology at http://www.epidem.com/). Information on the STROBE Initiative is available at www.strobe-statement.org.

For peer review only
